# Inactivation mode of sodium channels defines the different maximal firing rates of conventional versus atypical midbrain dopamine neurons

**DOI:** 10.1371/journal.pcbi.1009371

**Published:** 2021-09-17

**Authors:** Christopher J. Knowlton, Tabea Ines Ziouziou, Niklas Hammer, Jochen Roeper, Carmen C. Canavier

**Affiliations:** 1 Department of Cell Biology and Anatomy, School of Medicine, Louisiana State University Health Sciences Center, New Orleans, Louisiana, United States of America; 2 Institut für Neurophysiologie, Goethe University, Frankfurt, Germany; National Research Council, ITALY

## Abstract

Two subpopulations of midbrain dopamine (DA) neurons are known to have different dynamic firing ranges *in vitro* that correspond to distinct projection targets: the originally identified conventional DA neurons project to the dorsal striatum and the lateral shell of the nucleus accumbens, whereas an atypical DA population with higher maximum firing frequencies projects to prefrontal regions and other limbic regions including the medial shell of nucleus accumbens. Using a computational model, we show that previously identified differences in biophysical properties do not fully account for the larger dynamic range of the atypical population and predict that the major difference is that originally identified conventional cells have larger occupancy of voltage-gated sodium channels in a long-term inactivated state that recovers slowly; stronger sodium and potassium conductances during action potential firing are also predicted for the conventional compared to the atypical DA population. These differences in sodium channel gating imply that longer intervals between spikes are required in the conventional population for full recovery from long-term inactivation induced by the preceding spike, hence the lower maximum frequency. These same differences can also change the bifurcation structure to account for distinct modes of entry into depolarization block: abrupt versus gradual. The model predicted that in cells that have entered depolarization block, it is much more likely that an additional depolarization can evoke an action potential in conventional DA population. New experiments comparing lateral to medial shell projecting neurons confirmed this model prediction, with implications for differential synaptic integration in the two populations.

## Author summary

We developed a theoretical and mathematical framework that could explain the major electrophysiological differences between the conventional midbrain dopamine (DA) neurons with a low maximum firing rate, and the more recently identified atypical DA neurons. Testable predictions from this framework were then verified with *in vitro* patch-clamp recordings from DA neurons with identified phenotypes and projection targets. Since different subpopulations of DA neurons participate in different circuits, and these circuits are likely differentially dysregulated in diseases such as addiction, Parkinson disease, and schizophrenia, it is important to identify the differences of their intrinsic electrophysiological properties as a prelude to developing more precisely targeted therapies.

## Introduction

Midbrain dopaminergic signaling is strongly implicated in reward-based learning, motivation, action, and cognition [1–4]. The dopamine (DA) neurons in the substantia nigra pars compacta (SNc) and ventral tegmental area (VTA) are not a homogeneous population, instead, distinct DA subpopulations are differentially affected in major brain disorders such as Parkinson disease [5], schizophrenia [6] and drug abuse [7]. Dopaminergic signaling can only be understood by considering that dopamine neurons *in vivo* are not passive integrators of synaptic input. The intrinsic pacemaking activity exhibited by these neurons in a slice preparation is also observed *in vivo* [8,9], where it is additionally sculpted by synaptic inputs into less regular discharge patterns, including transient high frequency bursts and long pauses of electrical silence [8]. DA neurons in vivo fire at mean rates from 1–10 Hz, but bursts and pauses extend the dynamic range for the instantaneous firing rate from 0.5 Hz up to 100 Hz in some neurons [8]. Twenty percent of the neurons recorded in that study exhibited regular pacemaking activity at 2–4 Hz in awake behaving animals, thus the intrinsic activity of these neurons is important for their firing patterns *in vivo*. In this study, we start with the experimental observation that there are two electrophysiological phenotypes, conventional slow firing and atypical fast firing [10]. The goal of this study is to account for the contribution of intrinsic properties to this first major axis of diversity differences in the two populations identified our previous studies in order to better understand the contribution of subpopulations to the circuits in which dopamine neurons participate. Although the relative participation of these subpopulations in dopaminergic signaling is an unresolved, ongoing topic of investigation, an understanding of the biophysical basis for their intrinsic differences in firing range can lay the groundwork for understanding how the differences in dynamic range *in vivo* emerge.

Conventional midbrain dopamine neurons are associated with an electrophysiological profile that includes a slow firing rate, a broad action potential and a large sag current [11]. The maximal firing rate of midbrain dopamine neurons identified by these conventional electrophysiological features is limited to around 10 Hz [12] by abrupt entry into depolarization block. Studies that identified labeled dopamine neurons by projection target [10] revealed that the conventional population consists of midbrain dopamine neurons in the substantia nigra that project to the dorsal striatum, plus mostly lateral VTA and SNc neurons that project to the lateral shell of the nucleus accumbens. Moreover, a second population of dopamine neurons, projecting to the prefrontal cortex, amygdala and the core and medial shell of the nucleus accumbens and mostly located in the medial VTA, was found to have atypical electrophysiological properties. In contrast to the conventional population, this atypical population achieved maximal firing rates of 20–30 Hz prior to entry into depolarization block. During entry into depolarization block, a reduction in action potential (AP) amplitude was much more prominent in the atypical neurons compared to conventional ones. Depolarization block was first observed in dopamine neurons *in vivo* and was attributed to “inactivation of the spike generating mechanism” [13]. Voltage-gated sodium channels in dopamine neurons in the substantia nigra pars compacta (SNC) exhibit both fast and long-term modes of inactivation [14,15]. The long-term inactivated state is entered during brief depolarizing pulses, but recovers on a much longer time scale [16]. Here, we focus on the question whether the known differences between the two subpopulations can account for the difference in maximal firing rate and the different modes of entry into depolarization block, and on the sodium current as a potential key contributor to depolarization block. The differences in the intrinsic electrophysiological properties between these subpopulations may be important because different subpopulations of dopamine neurons participate in different functional circuits. For example, the atypical subpopulation appears to respond differently to aversive stimuli than the conventional neurons that signal reward prediction errors [17]. Understanding distinctions between subpopulations of dopamine neurons may be particularly relevant for designing pharmacological therapeutic interventions for disorders in which dysfunctional dopaminergic signaling is implicated, such as drug addiction [18,19] and schizophrenia [20]. Therefore, we used a simple mathematical model of a dopamine neuron to explain the biophysical basis for the differences between these two populations in dynamic range and mode of spike failure due to depolarization block. Novel model predictions were then experimentally tested via *in vitro* recordings from conventional and atypical DA cells identified by their projection targets.

## Results

### Sodium current calibration

Previous studies in midbrain dopamine neurons [21–24], like pioneering studies of long-term inactivation in hippocampal CA1 pyramidal neurons [25], used a formalism [26] with independent gates for activation, fast inactivation and slowly recovering inactivation. More recent work indicates that these processes are not independent, therefore we utilized a simple Markov model of this channel instead, modified from [27], in order to better capture the sodium channel dynamics crucial to the entry into depolarization block that limits the maximal firing rate. Since the majority of dopaminergic neurons prominently express Na_v_1.2 [28], with a small proportion expressing Na_V_1.6 [28], our model is based on Na_v_1.2 gating.

**Fig 1:**
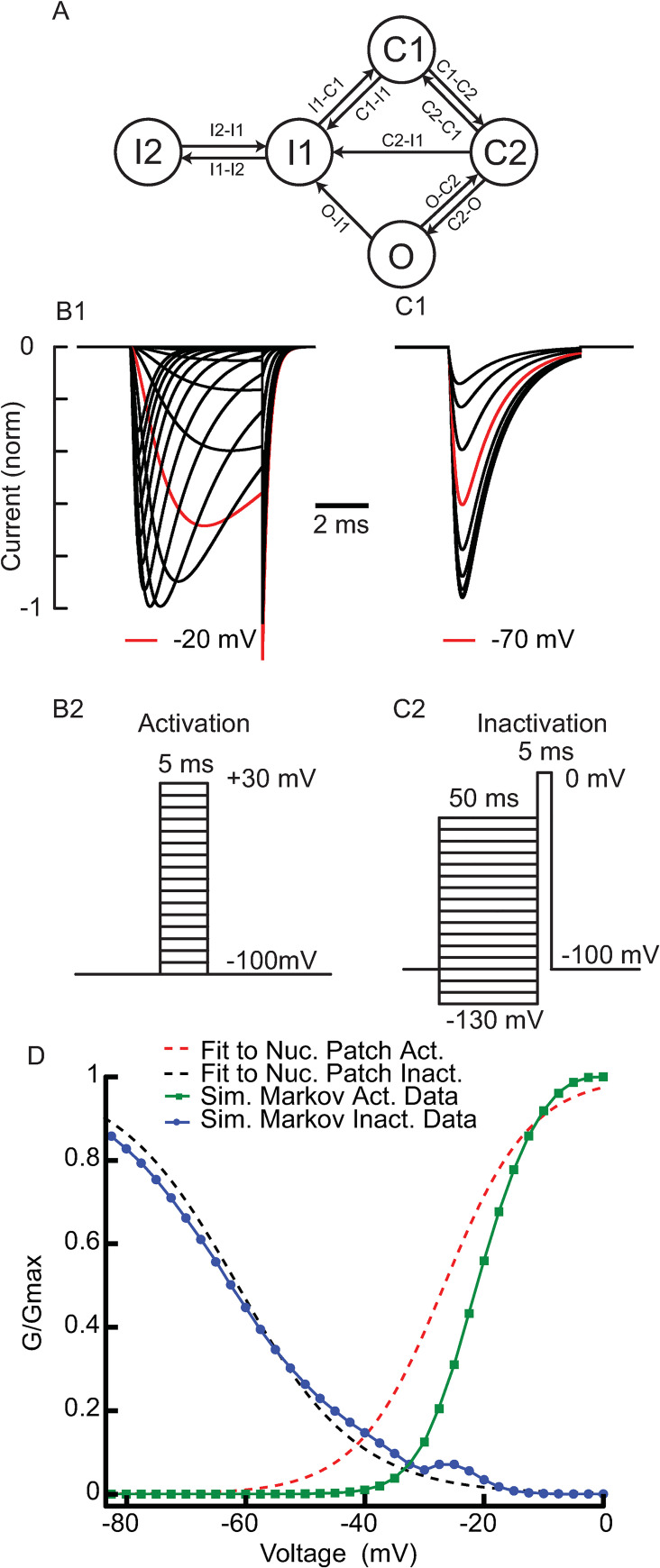
Calibration of Model Na_V_ 1.2. **A**. Markov model with 5 states for Na_V_ 1.2. Conductance is proportional to the open O1 state. C1 and C2 are closed states, I1 is the fast inactivated state and I2 is the long-term inactivated state. Voltage dependent rate functions for the transition rates that label each arrow are given in Table 1. The maximal I1-I2 transition rate was set to 26.7 s^-1^, but the voltage clamp protocol in the next panel is not sensitive to long-term inactivation due to the short duration test pulses and the lack of direct transition for O1 to I2 state. **B** and **C.** Simulated normalized model voltage clamp currents (top) using the protocols (bottom) of [15]. **B**. Activation. **C**. Fast inactivation. **D**. Simulated “steady state” dashed model activation curves compared to solid curves summarizing experimental data from [15]. All parameters are set to atypical values in Table 1.

The Markov model used for the sodium channels (Fig 1A) has an open state (O), two closed states (C1 and C2), and two inactivated states, a fast recovering (I1) and a long-term one (I2). The quantities above the arrows are the kinetic rates for the indicated transition. The “steady state” activation and inactivation curves for the model sodium current were initially calibrated using the protocols of [14,15] for activation (Fig 1B) and fast inactivation (Fig 1C), respectively. To estimate activation, the model was held at -100 mV then held at the test potential for 5 ms. The peak current divided by the Ohmic driving force at each potential, was used to calculate G/G_max_. To estimate inactivation, the model was held at -100 mV to remove most inactivation. Then a pre-pulse at each potential was applied for 50 ms. Next, the peak current during a 5 ms step to 0 mV was used to estimate inactivation, assuming again Ohmic drive and that the inactivation saturates during the 50 ms pre-pulse. Simulated “steady-state” points (Fig 1D) on the activation curve (green) and fast inactivation curve (blue) agree reasonably well with the Boltzmann functions that best fit the experimental data in nucleated patches from rat SNC dopamine neurons [14,15] for activation (black dashed curve) and inactivation (red dashed curve). The relatively depolarized action potential threshold in these neurons required a steeper [15], right-shifted model activation curve than observed in that study. This steeper activation curve is consistent with that reported in another study in the same cells [14]. Changes made to the original transition parameters for the Na_V_1.2 model from [27] (see Methods) increased the peak open fractions (>50%) in response to voltage clamp steps from hyperpolarized voltage, consistent with single channel data recorded from Na_V_1.2 channels [29].

One caveat is that although we have treated the Markov transition rates that determine the occupancy in the long-term inactivated state as parameters, *in vivo* they may be more analogous to state variables. Sodium channel availability is reduced by phosphorylation [30–32] at sites that regulate the long-term inactivation studied here as well as slow inactivation that requires seconds to develop [33]. Therefore, the neuromodulatory state of the neuron may affect transition rates.

### Basic mechanisms for limiting firing rate and entry into depolarization block

A previous study [10] characterized the electrophysiological differences between the conventional and atypical populations in part by their respective responses to triangular current ramps injected after hyperpolarizing the neurons to halt spontaneous pacemaking. Current ramps with a peak of 50 pA did not evoke depolarization block, whereas those with a larger peak current did. We will first focus on basic mechanisms for limiting the firing rate and entry into depolarization block. Once these are established, we will refine our preliminary models to better match the data and make experimental predictions.

**Fig 2:**
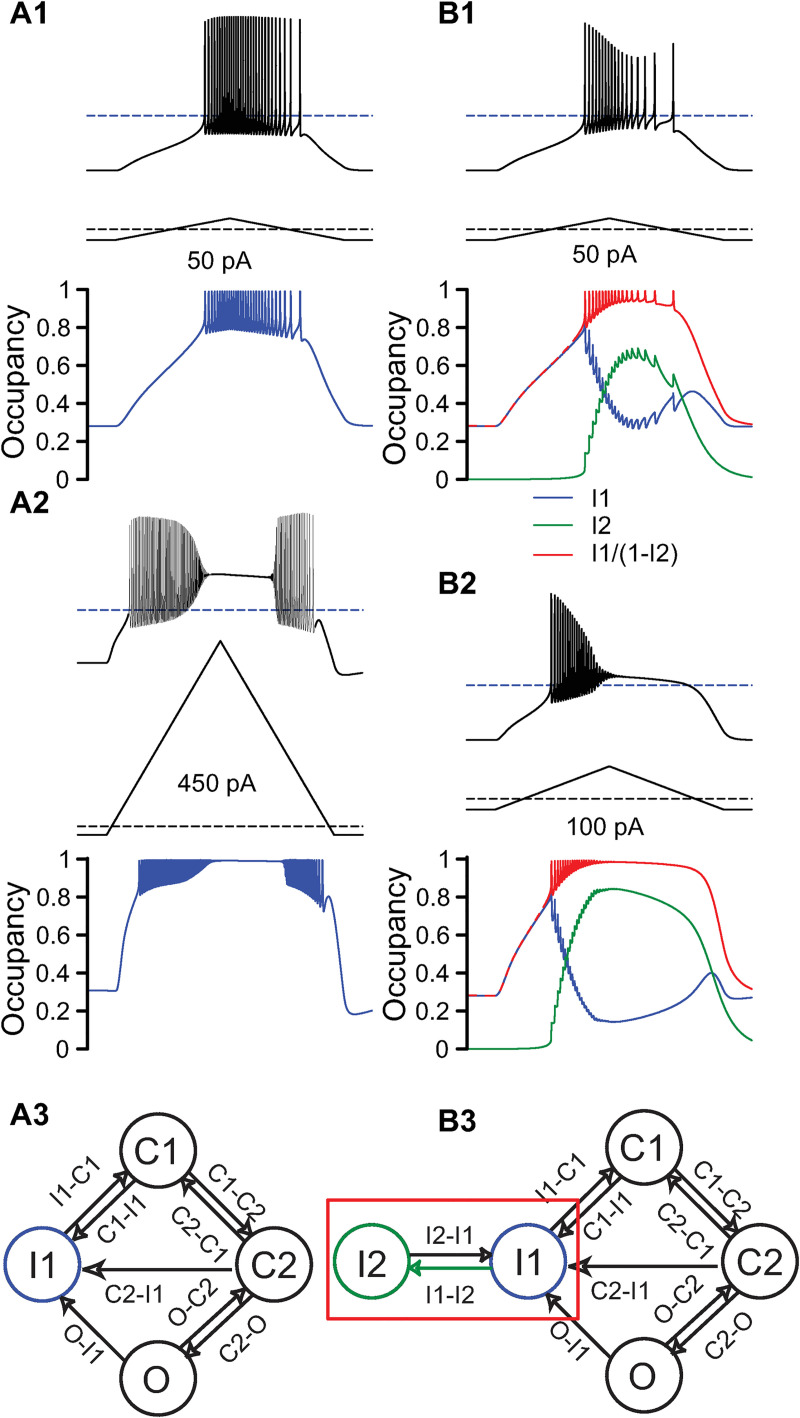
Atypical maximum frequency is limited by recovery from fast inactivation, but some long-term inactivation is needed for hysteresis. **A.** No long-term inactivation. **A1.** Weak triangular ramp (50 pA peak relative to baseline) in atypical model with no long-term inactivation (see panel A3) does not induce DP block. Time course of occupancy in the fast inactivated state, blue trace, bottom. **A2**. Strong triangular current ramp (600 pA) in atypical model with no long-term inactivation induces DP block with little hysteresis, such that spiking is reinstated on the down branch (membrane potential, upper trace). The fraction of channels in the fast inactivated state saturates (blue trace at bottom). **A3**. For I1-I2 = 0, the long-term inactivated state is effectively removed from the Markov model. **B**. Long-term inactivation added. The maximal I1-I2 transition rate is 26.7 s^-1^ as in Fig 1. **B1**. Response of atypical model (voltage trace, top) with long-term inactivation to a weak triangular current ramp (50 pA). **B2**. A stronger triangular ramp current (100 pA) induces depolarization block at a lower current amplitude than in **A2**. Bottom traces in each panel: time course of occupancy in I1 (blue) and I2 (green) states and their sum (red trace). **B3**. The green arrow in the Markov model shows which parameter was changed between **A** and **B**. Ramp currents are calibrated such that the initial hyperpolarizing step is identical for all cells (-25 pA) but the peak current relative to the hyperpolarized baseline is variable. Parameters are the ‘atypical’ values given in Table 1.

In Fig 2, we applied triangular current ramps, with the shape shown below each of the membrane potential traces, to an atypical model neuron calibrated to fit the data on the atypical population from [10] and with parameters as described in the Methods. Without occupancy in the long-term inactivation state (Fig 2A), a weak triangular current ramp did not induce depolarization block (Fig 2A1). Spiking does not cease due to depolarization block because a sufficient fraction of channels in the long-term inactivated state recovers between spikes (blue trace). However, the pattern of decreasing spike amplitude for the atypical population observed by [10] in their Fig 5B was not reproduced in the model with no long-term inactivation. A large triangular ramp (450 pA) was required to induce depolarization block on the up ramp; however, in contrast to the experimental data, depolarization block did not persist throughout the down ramp (Fig 2A2). The mechanism of depolarization block was that the occupancy in the fast inactivation state (bottom, blue trace) saturates to its depolarized steady state, which disables spiking, but as the ramp depolarization decreases, inactivation recovers rapidly so spiking resumes.

In the final version of the model of an atypical cell, we added a small amount of occupancy in the long-term inactivation state to prevent spiking on the down ramp after depolarization block as observed experimentally. In this case (Fig 2B1), a weak triangular ramp again did not induce depolarization block, but now a prominent reduction in spike amplitude was observed in the atypical model, consistent with experimental data. This is due to occupancy in the long-term inactivated state I2 (green trace) which causes the total number of inactivated channels (red trace) to accumulate between spikes during the ramp. Occupancy in the fast inactivated state (blue trace), from which channels quickly recover after an action potential, decreases during the ramp and correlates with decreased spike height. A much smaller triangular ramp (100 pA) was required to induce depolarization block in presence of long-term inactivation (Fig 2B2) than in its absence (compare to Fig 2A2). The maximum transition rate for I1-I2 in the atypical model was calibrated to match the peak firing rate at entry into depolarization block (20–30 Hz) of the atypical population in [10]. Depolarization block persists on the down ramp, and the action potential heights progressively decline in amplitude until they are very small at failure, consistent with the experimental data. Depolarization block persists on the down ramp because of the slow time course of recovery from the I2 state (green trace). The combined inactivation, I1+I2 occupancy (red trace), shows that consistent with the example with no long-term inactivation, depolarization block corresponds to a saturation of inactivation states.

**Fig 3:**
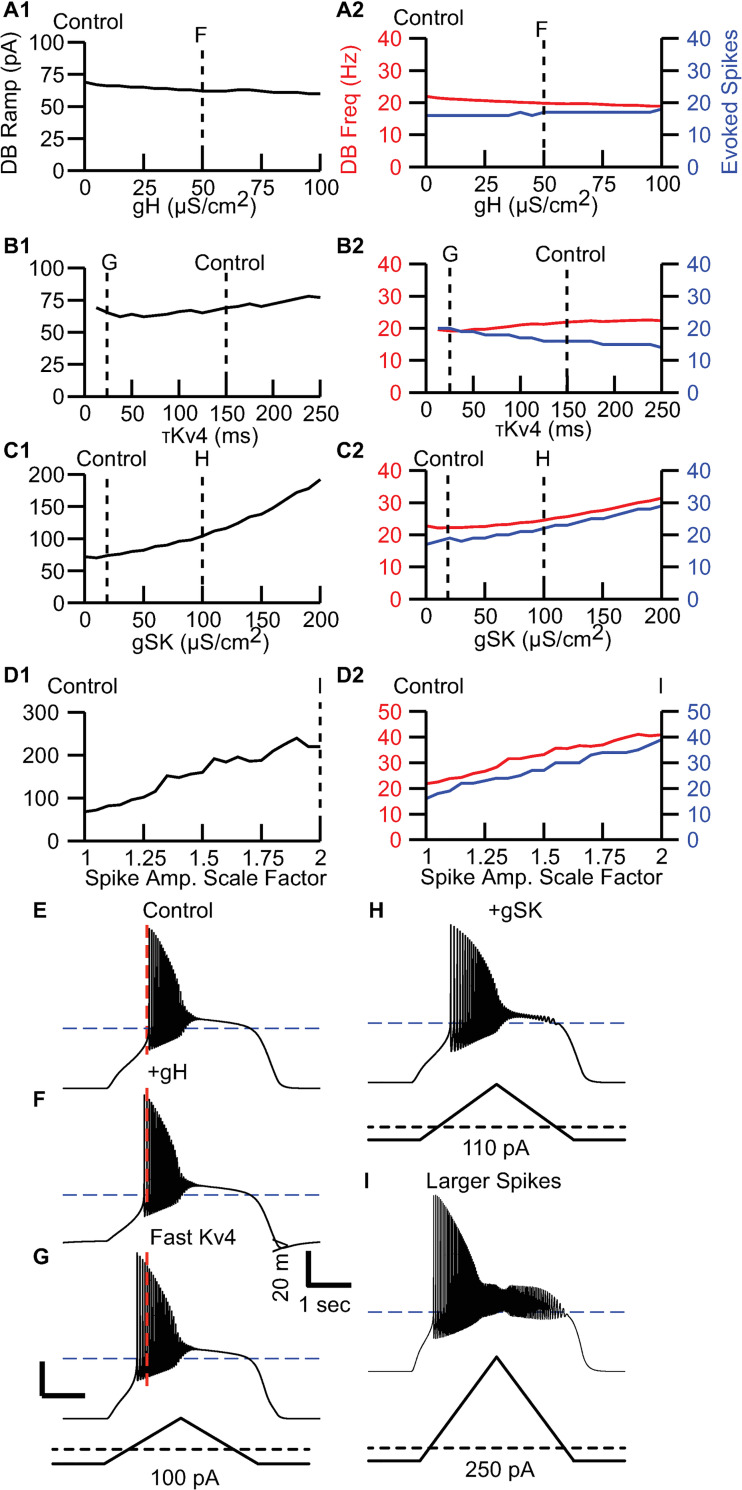
Known differences between atypical and conventional populations do not fully account for differences in peak firing rate or failure mode. **A**. Effects of varying maximal HCN conductance on A1, the minimum depolarizing current ramp amplitude required to induce depolarization block and A2, the peak firing rate (red) and number of evoked spikes (blue) on entry into depolarization block at the ramp amplitude from the previous panel. Dashed lines indicate values used for control and representative value in F-I. **A1**. **B.** Same as **A** except varying the Kv4 inactivation time constant. **C.** Same as **A** except varying the maximal SK conductance. **D** Same as **A** except varying the delayed rectifier and the sodium current by the same scale factor. **E**. Atypical control as in Fig 2B2 except in response to 75 pA ramp. Dashed line indicates time of first spike for control. Scale bar: 20 mV and 1 s. **F**. Increased H conductance. **G.** Faster Kv4.3 inactivation time constant. **H.** Elevated SK conductance in response to 110 pA ramp. **I.** Effect of increasing the primary spiking conductances (voltage gated sodium and the delayed rectifier) by an identical factor of 2 in response to 250 pA ramp. F. All parameters are set to atypical values in Table 1 except the parameter that is varied. The spike scale factor proportionally scales both gK,dr and gNa,V.

We next checked whether any of the known differences documented between the populations and listed in Table 1 contributed to limiting the maximum firing rate or to the mode of entry into depolarization block. Several differences in the biophysical properties between the subpopulations have been noted that might account for the different maximal rates and the different pattern of spike amplitudes upon entry into depolarization block. The sag amplitudes due to HCN channel activation are smaller in the atypical DA population compared to the conventional one [10]. However, this conductance (g_H_) had only a minor effect on the amplitude of the current ramp required to induce depolarization block (Fig 3A1) and a negligible effect on the maximum frequency (red curve Fig 3A2) that can be achieved prior to depolarization block. The blue curves showing the number of spikes during the ramp are provided to show that quite a few spikes are evoked prior to DP block, as expected near the threshold for depolarization block. The time constant (τ_kv4_) of inactivation of Kv4.3 channels is more than five-fold slower in the atypical population compared to the conventional population [34,35]. However, varying this time constant, which also controls the rate of recovery from inactivation within the physiological voltage range also had a very small effect on the current required to induce depolarization block (Fig 3B1), and again a negligible effect on the maximum frequency (red curve Fig 3B2) that can be achieved prior to depolarization block. The AHP is also reduced in the atypical versus conventional cells [10], consistent with previous results showing lower expression of the Ca^2+^-activated small conductance potassium channel SK3 in the VTA as compared to the SN [36]. SK channels are a major contributor to the medium AHP in SN DA neurons [37], thus in Fig 3C we varied the SK conductance (g_SK_). Increasing this conductance increased the amount of current required to drive the model neuron into depolarization block (Fig 3C1), and concomitantly increased the maximum frequency (red curve Fig 3C2) that can be achieved prior to depolarization block. Since this conductance is greater in the conventional population, and the conventional population has a lower maximal frequency, this change is in the wrong direction, so the difference in SK conductance cannot be responsible for the lower maximum firing rate in the conventional population. Action potential amplitudes were smaller in the fast-spiking population (this is addressed in detail later in the Results section when the phase plots of the populations are compared). From this, we conclude that those spiking conductances that are associated with the fast, voltage-gated sodium current (g_NaV_) and the delayed rectifier potassium current (g_Kdr_) are larger in the conventional population. In order to maintain normal spiking, it was necessary to co-vary those conductances, so we adjusted them both by the same scale factor in Fig 3D. Again, the tendency is in the wrong direction, with the stronger spiking conductances that characterize the conventional population leading to higher maximum frequencies, contrary to experimental observations. Therefore, we conclude that none of the currently defined biophysical differences between the populations do account for the slower maximum rates.

**Table 1:** Differences between conventional and atypical exemplar models. The differences in parameters are based on the literature and explained in the text for Fig 3, except the difference in the maximal transition rate from the fast to the long-term inactivated state, CI1I2, which is based on Fig 5.

	Atypical	Conventional
g_H_	0 μS/cm^2^	50 μS/cm^2^
τ_kv4_	150 ms	25 ms
g_Kdr_	1250 μS/cm^2^	2500 μS/cm^2^
g_NaV_	15 mS/cm^2^	30 mS/cm^2^
g_SK_	20 μS/cm^2^	100 μS/cm^2^
CI1-I2	26.7 s^-1^	100 s^-1^
Length	500 μm	1000 μm

Finally, in [10], we also found that the atypical and conventional cells formed separate clusters in a space of seven electrophysiological variables, including membrane capacitance as a readout of membrane surface area. Although not explicitly stated in that study, the conventional cluster consisted of larger cells than the atypical cluster based on capacitance measurements. In a one compartment model, changing the surface area proportionally scaled the amount of current required for entry into depolarization block and nothing else, so size alone is likely not a factor in changing the maximum observable frequency.

Fig 3E repeats the panel in Fig 2B2 and illustrates entry into depolarization block by the model atypical neuron. Fig 3F shows that increasing the conductance g_H_ for the HCN channel that mediates Ih current to match that of the conventional model only causes spiking to commence slightly sooner. Decreasing the time constant of recovery from inactivation of Kv4.3 in Fig 3G allows spiking to begin noticeably sooner. Increasing the SK conductance in Fig 3H evokes additional sustained outward current that partially counteracts the depolarizing ramp current, and thus requires additional current to induce depolarization block [38]. Spiking begins earlier on the ramp due to the larger depolarizing current amplitude, and the transition to depolarization block is even more gradual, but clearly increasing the SK conductance does not make the atypical model more conventional. In addition to slightly increasing the sustained outward current, the elevated delayed rectifier conductance removes inactivation more effectively right after each spike, so stronger current ramp amplitudes can be tolerated before going into depolarization block (as explained in the next section). Fig 3I shows that increasing the spiking conductances requires a much stronger depolarizing current ramp to induce depolarization block largely because sodium channel inactivation is more effectively removed during the AHP. The subthreshold oscillations observed after entry into depolarization block are more prominent, but again, increasing the spiking conductances does not make the atypical model more conventional. Since no known individual parameter difference made the atypical more conventional, and in fact in two of the four cases went in the opposite direction, it is unlikely that any combination of parameter changes from this set could convert the atypical model into a conventional one.

**Fig 4:**
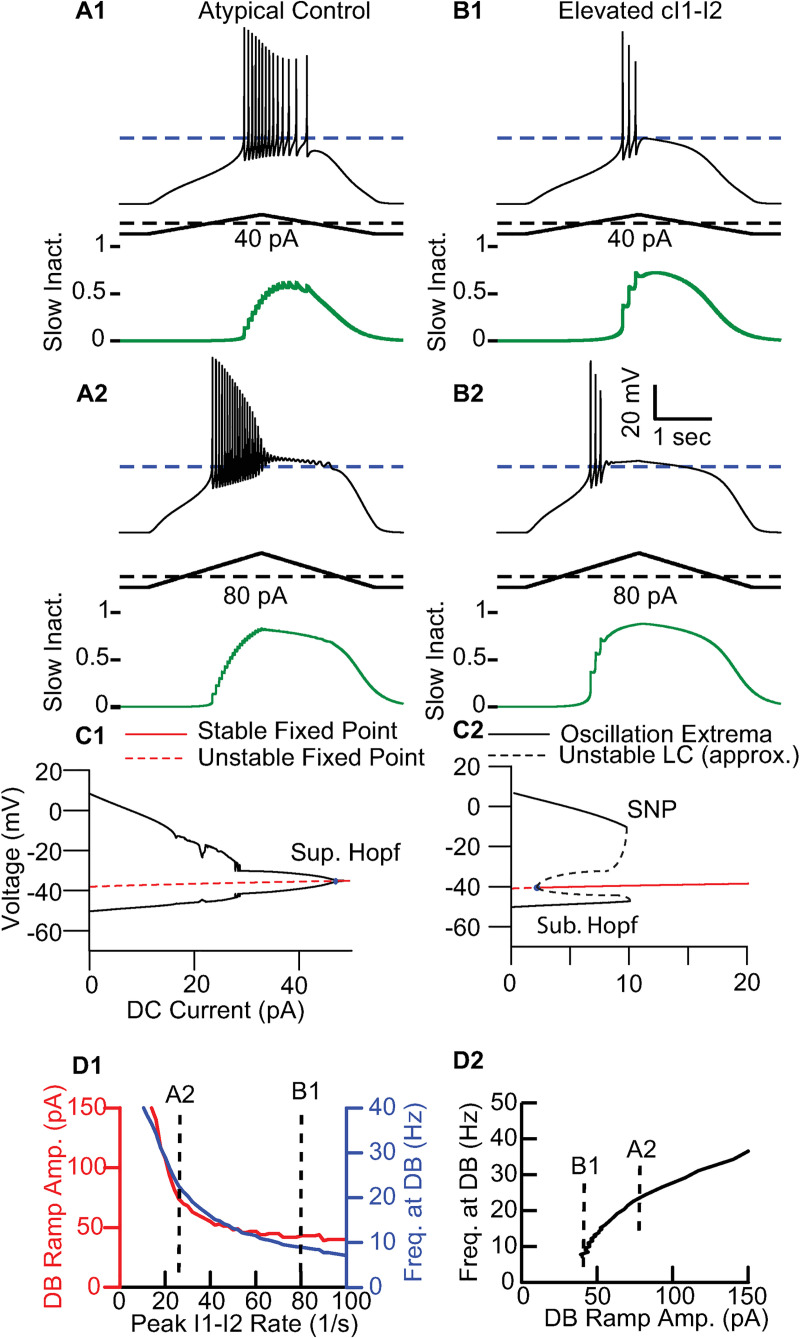
Additional Long-term Inactivation Converts Atypical to Conventional Failure. **A**. Atypical Model. During ramp stimulation, each spike produces a small uptick in occupancy in the long-term inactivated state I2 (green curves) **A1**. A 40 pA ramp does not induce depolarization block in the atypical model. **A2**. An 80 pA ramp results in depolarization block in the atypical model. **B**. Atypical Model with increased occupancy in the slowly inactivated state. **B1**. Increasing the maximum rate of the I1 to I2 transition from 26.7 s^-1^ to 80 ms^-1^ leads to failure at substantially lower frequencies for 40 pA ramp current levels. Large upticks in the level of slow inactivation (green trace) are observed for each spike. **B2**. A larger ramp with peak amplitude of 80 pA, beyond that required to induce depolarization block. **C**. Bifurcation diagrams with respect to applied current. **C1**. Atypical model. Oscillations terminate via supercritical Hopf. **C2.** Elevated cI1-I2. Oscillations terminate via saddle node of periodics (SNP) following subcritical Hopf where the unstable limit cycle (LC) originates. **D**, Effect of I1-I2 transition rate on entry in depolarization block. **D1**. Dependence of the minimum ramp amplitude required to induce depolarization block on the I1-I2 transition rate (red curve) and the frequency upon entry into depolarization block (blue curve). For the atypical value of 26.7 s^-1^ for the maximum I1-I2 transition rate, an 80 pA peak ramp amplitude is sufficient to induce depolarization block with a maximum frequency of about 23 Hz (vertical dashed line labeled A2). Increasing the I1-I2 maximum transition rate to 80 s^-1^ drops the required peak ramp amplitude to 50 pA and the maximum frequency to about 13 Hz (vertical dashed line labeled B1). **D2**. The larger the peak ramp current tolerated prior to entry into depolarization block, the larger the maximum frequency observed during the ramp. All parameters are set to atypical values in Table 1 except C_I1-I2_, which is variable.

Since none of the known differences did account for the much slower maximal firing rate of the conventional population, we hypothesized that occupancy in the long-term inactivated state rather than the fast inactivated state of the sodium channel might limit the firing rate in the conventional population. We chose the maximum I1-I2 transition rate to manipulate occupancy in the I2 state. Fig 4 systematically assesses the effect of the maximum I1-I2 transition rate on the ramp amplitude required to induce depolarization block and upon the maximum instantaneous frequency (the reciprocal of the last ISI) prior to entry into depolarization block. Consistent with the 50 pA ramp in Figs 2B1 and 4A1 shows that a ramp amplitude of 40 pA is insufficient to induce depolarization block. Fig 4A2 shows that a ramp of 80 pA is sufficient, and that each spike causes only a small increment in I2 occupancy (green trace). As I2 occupancy accumulates, each successive spike has a smaller amplitude, until the final spike before failure is quite small. In Fig 4B, the kinetic rate for the transition between the fast-inactivated state (I1) and the long-term inactivated state (I2) was increased (from 26.7 /s to 80 /s) for a very preliminary prototype of a conventional cell. This single parameter change led to failure (Fig 4B1) at substantially lower ramp current levels (40 pA) that do not produce depolarization block with the lower transition rate. Failure at lower currents dropped the maximum frequency prior to entry into depolarization block from 20 to 9 Hz. Failure was more abrupt in this case; each spike now resulted large upticks in the level of long-term inactivation (green trace) such that the last large uptick resulted in a failure to initiate spiking. In Fig 4B2 a larger ramp with peak amplitude of 80 pA, beyond that required to induce depolarization block slightly increased the maximum frequency and pushed the point of failure earlier in the ramp. Fig 4C1 and 4C2 shows the respective bifurcation diagrams for the atypical model and the model with increased occupancy in the long-term inactivated state, with constant applied current as the bifurcation parameter. Values of the membrane potential at which the net ionic current is zero are indicated in red and called fixed points. If the fixed point is stable, this is the holding potential at a given level of applied current. We will refer to this holding potential as the “resting potential” at a given level of applied current, whether it is stable or not. Oscillatory solutions are indicated in black and only the maxima and minima are plotted such that an oscillatory solution has two branches in the bifurcation diagram. For the atypical cell model, the action potential threshold is consistently below the (unstable) “resting” membrane potential, allowing oscillations to persist at gradually decreasing amplitudes until the entire pool of available sodium channels is exhausted, consistent with a supercritical Hopf bifurcation to terminate spiking (see Fig 4C1) In the modified model from Fig 4B, increased occupancy in the I2 state causes the action potential threshold to be more depolarized than the “resting” potential. This stabilizes a fixed point at the resting potential “inside” the oscillatory solution (limit cycle) that corresponds to periodic spiking, consistent with a subcritical Hopf (see Sub. Hopf in Fig 4C2). An unstable limit cycle (dashed black branches) arises from the Hopf bifurcation with a smoothly varying amplitude, but only stable oscillations are observable in the physical world due to noise. The abrupt failure of spiking results from a saddle node of periodics (SNP) when the stable pacemaking limit cycle (solid black branches) collides with the unstable limit cycle (dashed black branches), abolishing spiking with large AP amplitudes. See also S1 Fig for additional details.

Fig 4D1 shows that as the maximum I1-I2 transition rate was increased, both the amount of current that was tolerated prior to entry into depolarization block and the maximum frequency observed prior to depolarization block steadily decrease. Fig 4D2 depicts the co-dependence of maximum frequency and maximum ramp amplitude.

Although Fig 4 demonstrates that variations of long-term inactivation made a large contribution to the distinct dynamics between the populations, it cannot be the only difference. There are likely at least two other types of differences between the populations that offset the tendency of additional long-term inactivation to go into depolarization block with smaller current ramps. The first is the difference in size between the populations; the atypical population has smaller neurons. The ramp current is given in extensive units of current, but the single compartment model is formulated in terms of intensive units of current per unit area. Therefore, doubling the surface area doubles the size of the current ramp required to achieve the exact same effect. Henceforth, we assigned a larger surface area (greater length in Table 1) to the conventional population to increase the current required to go into depolarization block to values comparable to those for the atypical population. Moreover, the deeper AHP in the conventional population partially counteracted the effect of the increase in depolarizing driving current, allowing spiking to continue at higher levels of injected ramp current. Increasing AHP depth via increases in the delayed rectifier only affected the gain of the cells, causing failure at higher driving currents, but identical frequencies. However, the AHP mediated by SK channels persisted longer and increased the maximal frequency at failure. Thus, some combination of deeper and/or longer AHP and larger surface area increases the size of the current ramp required to induce depolarization block in conventional cells to a level comparable to atypical cells, in addition to the hypothesized larger contribution of long-term inactivation in conventional cells. These differences were incorporated into a calibrated conventional cell model in a principled way as described below.

**Fig 5:**
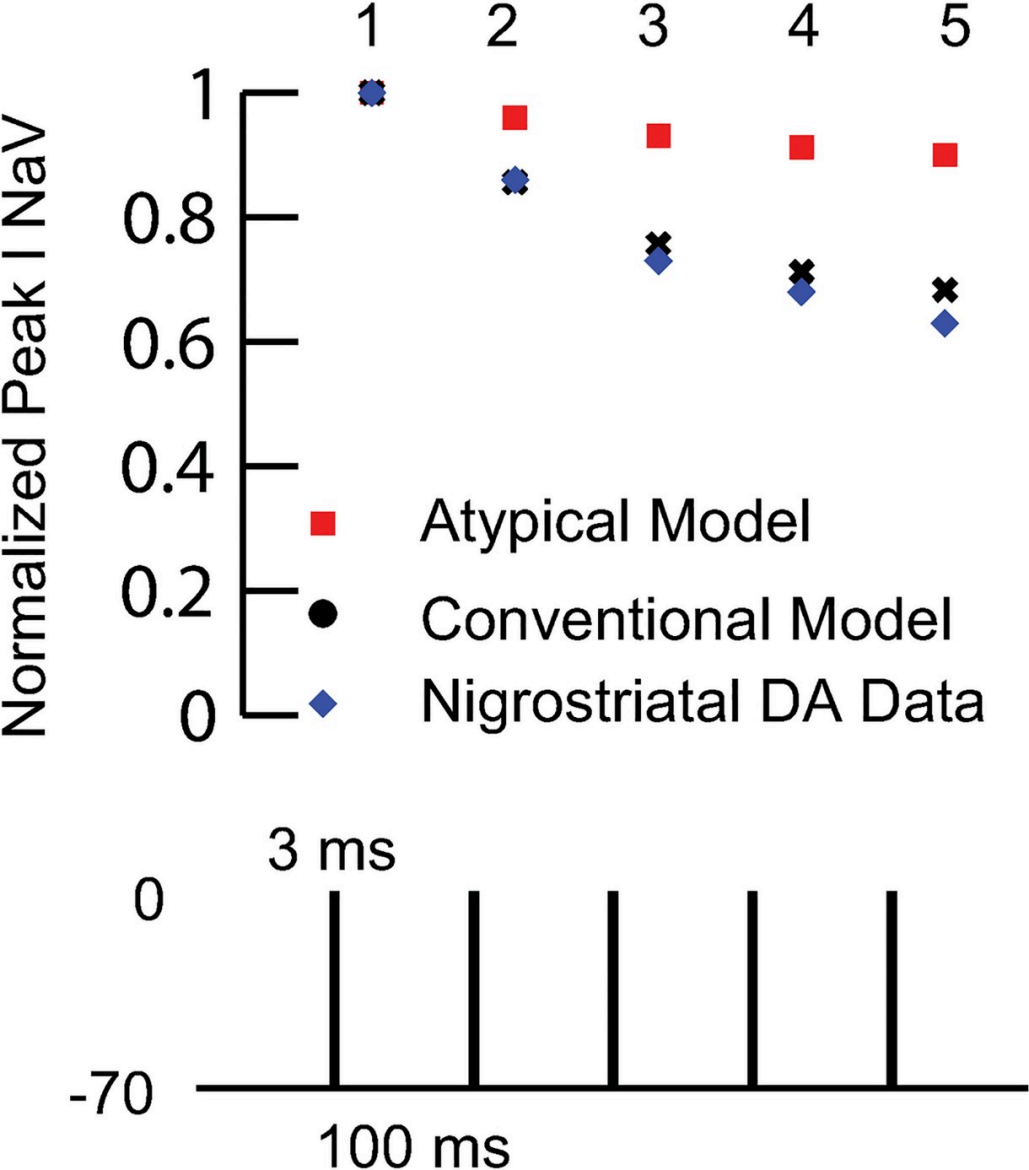
Calibration of Long-term Sodium Channel Inactivation of Conventional Model Dopamine Cells Compared to Atypical Model. Fit of long-term inactivation to data. The protocol of [15] consisting of five brief voltage clamp steps was used to calibrate the conventional model (black dots) and compare to the data taken from [15] (blue diamonds). The red squares are the prediction for the atypical cells. The maximum rate of the I1 to I2 transition was increased to 100 s^-1^ for conventional from 26.7 s^-1^ for atypical.

The amount of long-term inactivation of the sodium channel required for the atypical model was determined in Fig 2B. Fig 5 shows that the time course of entry of the conventional model into the long-term inactivated state (black circles), calibrated according to experimental protocol [15], is a reasonable match to the published data (blue diamonds). The response of the atypical model (red squares) to the same experimental protocol is a prediction based on Fig 2B, because this quantity has not yet been measured in the atypical population. After establishing the parameters for long-term inactivation in the conventional model, we proceeded to calibrate other electrophysiological properties.

**Fig 6:**
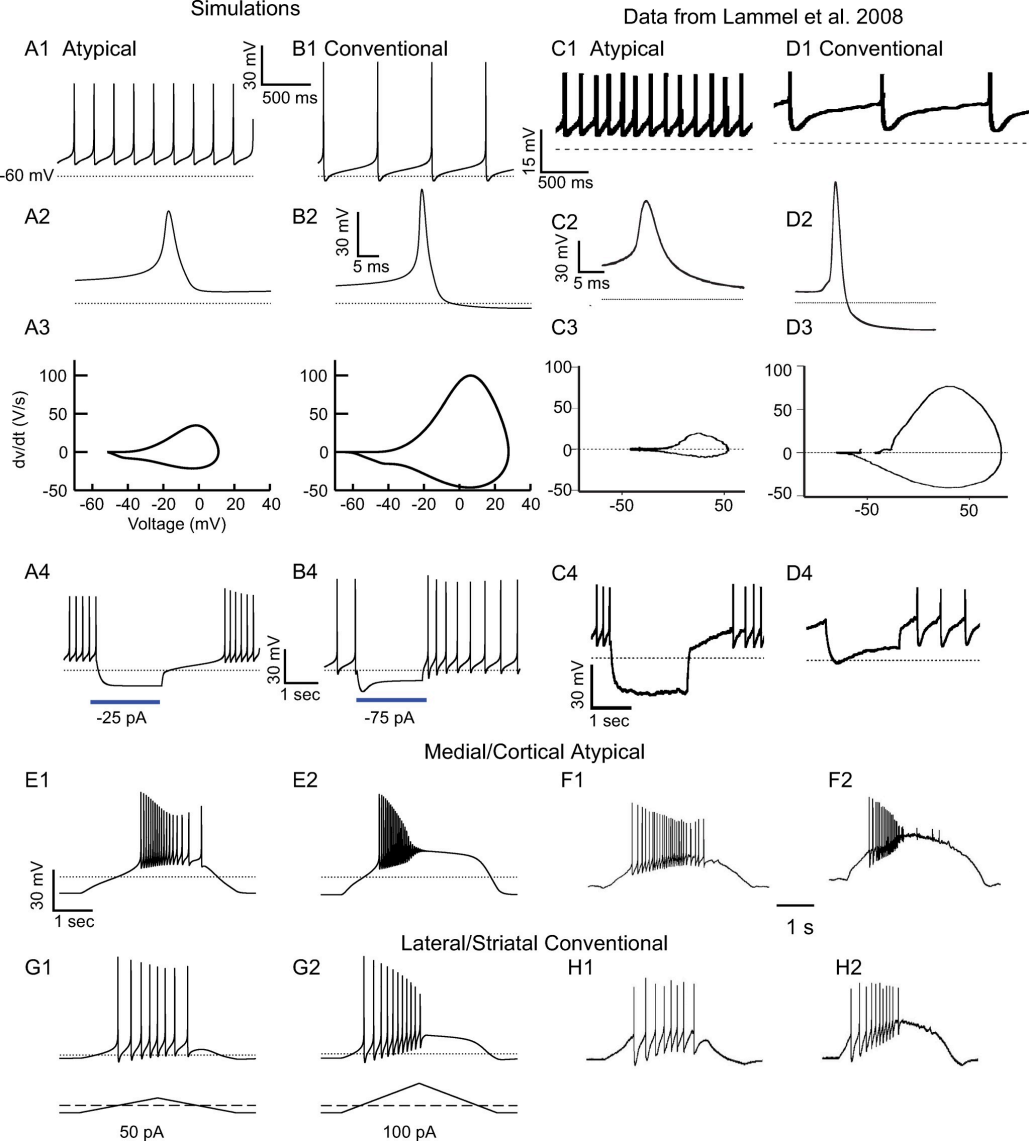
Calibration of Electrophysiological Profile of Atypical and Conventional Model Dopamine Cells Compared to Experimental Data from the Literature. **A.** Atypical Model. **A1.** Spontaneous pacemaking at 5 Hz. **A2.** Single action potential from **A1** with a peak of 11 mV, width of 5 ms at -30 mV, and minimum AHP depth of -51 mV. **A3.** Phase plot of dV/dt vs V. **A4.** Response to a 2 s, 25 pA hyperpolarization. **B.** Conventional model. **B1.** Spontaneous pacemaking at 2 Hz**. B2.** Single action potential from **B1** with a width of 3 ms at -30 mV, peak amplitude of 28 mV and minimum AHP of -64 mV. **B3.** Phase plot of dV/dt vs V. **B4.** Response to a 2 s, 75 pA hyperpolarization. **C1-C4.** Same protocol as A1-A4 except recorded from an identified mesocortical VTA atypical dopamine neuron. **D1-D4.** Same protocol as **B1-B4** except recorded from an identified mesostriatal SN conventional dopamine neuron**. E- H,** Responses from a hyperpolarized, silent state to depolarizing current ramps. **E.** Atypical model. **E1.** Response to 50 pA triangular ramp. **E2.** Response to 100 pA ramp. **F1-F2**. same protocol as **E1-E2** as except recorded from an identified mesolimbic medial shell projecting atypical dopamine neuron. **G.** Conventional model. **G1.** Response to a 50 pA triangular ramp. **G2.** Response to 100 pA triangular ramp. **H1-H2**. same protocol as G1-G2 except recorded from an identified mesostriatal projecting conventional dopamine neuron. **C, D, F and H** were modified with permission from Figs 4A–4D and 5B and 5C in the complete Elsevier source [10].

The electrophysiological properties of the atypical model from Fig 2A and 2B were previously calibrated according to published data (Fig 4D in [10]) to capture the spontaneous pacemaking rate (Fig 6A1), the single action potential shape (Fig 6A2) and the phase plane representation (Fig 6A3) that plots the first temporal derivative of the action potential membrane potential versus the membrane potential. The conventional model was calibrated in a similar manner in Fig 6B. Consistent with the ranges given in [10], the atypical model had a faster spontaneous pacemaking rate than the conventional model (3.5 Hz vs 1.5 Hz), compare Fig 6B1 to 6A1. The parameter changes in Table 1 converted the smaller, wider action potential in the atypical model to the larger but narrower action potential emitted by the calibrated conventional model, compare Fig 6B2 to 6A2. Consistent with our published data [10] reproduced in Fig 6C3 and 6D3, conventional model cells have much larger rise and fall rates of atypical cells, compare Fig 6B3 to 6A3. Finally, also consistent with Fig 4 from [10] and reproduced with permission in Fig 6C4 and 6D4, the atypical model showed no sag potential and a longer rebound delay in response to hyperpolarizing step currents (Fig 6A4), whereas the conventional model exhibited a sag potential and a faster rebound (Fig 6B4). The bottom two panels of Fig 6 make a head-to-head comparison of the responses of the atypical model (Fig 6E, repeated from Fig 2B) and the calibrated conventional model (Fig 6G) to ramp amplitudes comparable to those applied experimentally in Fig 5 of [10] and reproduced with permission in Fig 6F and 6H. We previously showed (Fig 2B) that the atypical model captures the essential aspects of the atypical ramp responses. Here we show that the conventional model also captures the essential aspects of the conventional ramp responses. For the weaker ramp in Fig 6G1, the evoked firing rate varies between 3–5 Hz with spikes of consistent magnitude and AHPs with depths comparable to experimentally observed ones that initially go below the post sag baseline established by the -25 pA pre-ramp holding current. Moreover, the number of spikes on the ascending part of the ramp (6) is greater than on the descending ramp (2), capturing the hysteresis. For the stronger ramp in Fig 6G2, spiking fails at an instantaneous frequency of 9 Hz prior to the ramp peak, and the last spike has considerable amplitude. (See S1 Fig for the bifurcation diagram for the exemplar conventional model). The increased spiking conductances plus doubling the surface area and increased SK conductances of the conventional model prevented depolarization block until the current ramp reaches 100 pA (lower trace). Therefore, the model suggests that multiple differences between the subpopulations are required to account for their distinct electrophysiological profiles.

**Fig 7:**
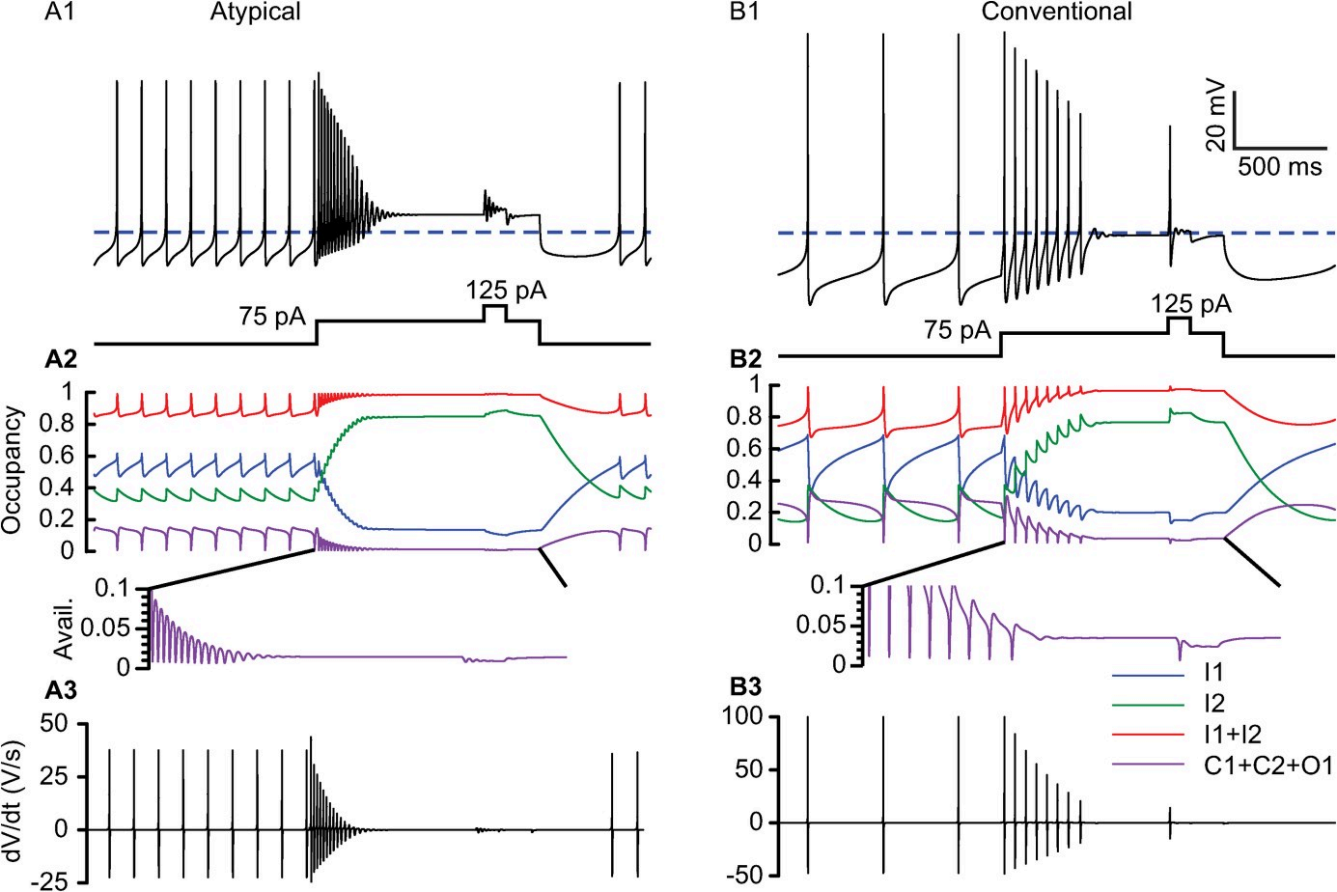
Explanation of Depolarization Block Mechanisms Using Square Pulses. A. Atypical model **A1**. Voltage traces (top) of a spontaneously pacing model neuron in response to a 2 sec, 75 pA square current pulse, with an additional 200 ms 50 pA step (bottom) applied after the model enters depolarization block. Depolarization block is entered via a gradual decrease in spike amplitude, with a maximum firing rate of 28 Hz. An additional current step during depolarization block does not evoke additional spikes. **A2**. Time course of available sodium channels (purple) (C1+C2+O1). Time course of occupancy in I1 (blue), I2 (green), and their sum (red). Inset: Low available pool prevents large oscillations following entry into depolarization block. **A3.** dV/dt for voltage trace in A1. Oscillations rapidly fall below the spike threshold (5 V/s), and do not pass above that point in response to added current. **B**. Conventional cell model. As in the previous figure, the conventional model has twice the surface area as the atypical. Voltage traces (top) of a spontaneously pacing model neuron in response to a 75 pA step current with an additional 200 ms, 50 pA step following entry into depolarization block. Depolarization block occurs abruptly at 10 Hz with a large amplitude spike, but an additional spike can be evoked with additional current. **B2**. Time course of available sodium channels (C1+C2+O1) (purple). Occupancy in I1 (blue), I2 (green), and their sum (red) for voltage trace in B1. Inset: relative to B2, larger fractions of sodium channel remain available for spikes evoked by additional current. **B3.** dV/dt for voltage trace in B1. The action potential evoked by additional current has peak slope well above spike threshold.

In Fig 7, we examine entry into depolarization block by using square pulses applied during spontaneous pacemaking, rather triangular depolarizing pulses applied after a hyperpolarization that silences the neuron as in Figs 2B2, 4B, 5E–5H and 6E2, 6F2, 6G2, and 6H2. In the interest of better characterizing the underlying mechanism, we used longer (2 s) depolarizing step currents, compared to previous experimental studies [12], to allow transients arising from the slow kinetics of the recovery from long-term inactivation of the sodium channel to reach a steady state. In all panels, equilibrated spontaneous pacemaking was established before the square pulse was applied. As shown in Fig 7A1, the atypical model response went into depolarization block during a 75 pA square current pulse in a manner similar to that observed during a ramp; the action potentials gradually decline in amplitude until they fail. This is a model prediction because we are not aware of previously published data on the response of atypical cells to square pulses. During spontaneous pacemaking prior to the pulse, Fig 7A2 shows that the increase in occupancy in the long-term inactivated state (green) evoked by each action potential was matched exactly by the amount of decay between action potentials. Similarly, the amount of occupancy in the fast inactivated state (blue) that accumulates during the preceding interspike interval and action potential was exactly removed during the AHP. During the depolarizing current step, the interspike interval became too short to remove all the long-term inactivation induced by the preceding action potential, and the AHP became insufficiently deep to remove the accumulated fast inactivation due to the preceding spike and interspike interval. Depolarization block occurs when the vast majority of the channels are in one of the two inactivated states (red). An additional 50 pA step of depolarizing current failed to evoke an action potential because the available pool of sodium channels that were not in one of the two inactivated states (purple, see also magnification in Fig 7A2) was essentially frozen after the last spike prior to entering depolarization block. Thus, any additional spike evoked by additional depolarization was unlikely to exceed the amplitude of the last spike before failure. The capacitive current was approximated by the derivative of the membrane potential waveform in Fig 7A3.

The conventional model sustained pacemaking in a similar manner to the atypical model (Fig 7B1). A current step of 75 pA induced depolarization block in a manner similar to that observed in the ramps and in the experimental data for square pulses [12]; the spike amplitude decreased slightly, but the last spike prior to failure was still fairly large. As with the ramps, each spike produces a larger increment in the I2 state (Fig 7B2, green) compared to in Fig 7A2. Compared to the atypical models, a lower proportion of channels were in the either of the two inactivated states when depolarization block occurred (purple, see magnification in Fig 7B2). Notably, an additional depolarizing step applied after depolarization block evoked an additional action potential. The ability of the conventional model to fire large amplitude spikes from a depolarization block state directly follows from the abrupt failure after a large amplitude spike, allowing a sufficient remaining pool of sodium channels available to produce a regenerative event when recruited by additional depolarization. The abrupt failure results from a saddle node of periodics bifurcation, as explained in the text for Fig 4C2 and illustrated for the conventional model in S1 Fig.

**Fig 8:**
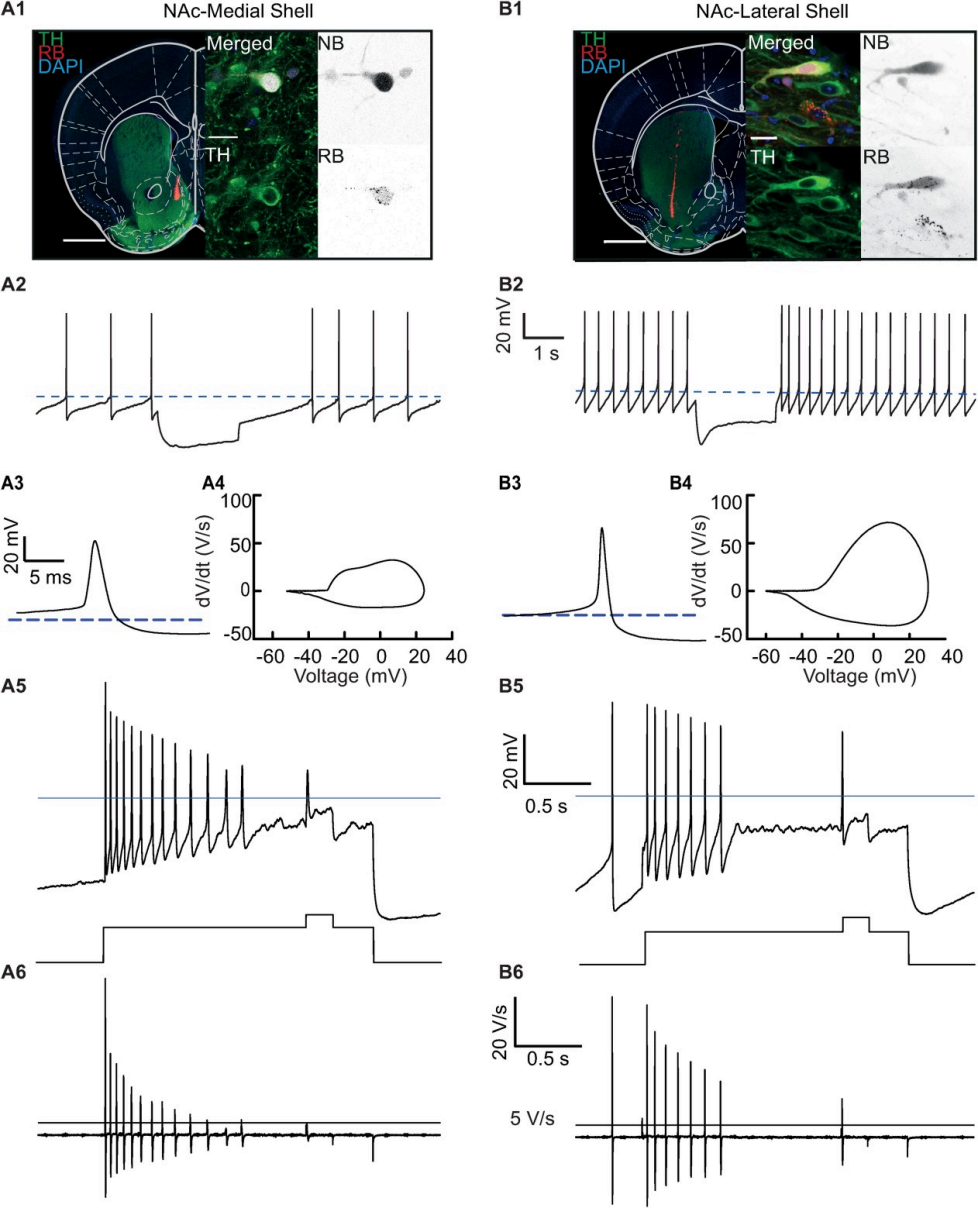
Response of midbrain dopamine cells with identified projection targets to additional depolarization after inducing depolarization block with a current step. **A1, B1.** Double labeling of cells with TH (tyrosine hydroxylase) to confirm dopaminergic phenotype and retrobeads (RB) to confirm projection target is medial shell (A1) or lateral shell (B2). **A2, B2.** Rebound response of identified cells to 2s of hyperpolarizing current. Cell from A1 does not show significant sag potential and has a 1.9 s rebound delay, confirming atypical phenotype (A2). Cell from B1 has a post sag potential of +19 mV and a rebound delay of 145 ms, consistent with conventional phenotype (B2). **A3.** Action potential waveform during pacing of identified medial shell projecting cell from A1. Action potential threshold is -30 mV and width at threshold is 5.2 ms. **A4**. Phase plot (slope vs voltage) of action potential in **A3**. Peak slopes are +30 and -18 V/s. **B3.** Action potential waveform during pacing of identified lateral shell projecting cell from B1. Action potential threshold is -27 mV and width at threshold is 3.7 ms. **B4**. Phase plot of action potential in B3. Peak slopes are +70 and -33 V/s. **A5**. Voltage trace of entry into depolarization block in response to 2s 125 pA pulse with an additional 200 ms pulse of 75 pA after 1.5 s for medial projecting cell identified in A1. Spike amplitude decays continuously into near threshold oscillation with a peak frequency (avg of first 3 ISI) is 40.5 Hz and a terminal frequency (average of final 3 ISI) of 20.7 Hz. Additional current pulse evokes a 20 mV amplitude oscillation. **A6**. Plot of dV/dt for voltage trace in A5. Slope declines continuously to below 5 V/s spike threshold. Peak slope of evoked oscillation is 2.6 V/s. **B5**. Voltage vs time in response to 2s 300 pA pulse with additional 200 ms pulse of 75 pA after 1.5 s in lateral projecting cell identified in B1. Spike amplitude remains consistent with a peak frequency of 15.7 hz and terminal frequency of 8.7 Hz. Additional current pulse evokes a 44 mV amplitude spike. **B6**. Plot of dV/dt for voltage trace in B5. Slope declines from spike to spike with final spike having a peak amplitude of 25 V/s. Evoked spike has a peak slope of 18 V/s, well above spike threshold of 5 V/s.

In order to test the novel predictions of our models shown in Fig 7, we applied similar stimuli to identified conventional and atypical DA neurons, which were doubled labeled with tyrosine hydroxylase (TH) to confirm dopaminergic phenotype and with retro beads (RB) injected into respective projection areas, either the medial shell of the nucleus accumbens for atypical DA neurons (Fig 8A1) or the lateral (Fig 8B1) shell of the nucleus accumbens for conventional DA neurons. The electrophysiological phenotype of the identified DA neurons was confirmed by their pacemaker activity and the presence of a pronounced sag current in the lateral but not medial projecting cells (Fig 8A2 and 8B2), as well as a longer latency to the first action potential after a hyperpolarization in the medial projecting cells. Single action potentials recordings and phase plane plots are given for an example ‘atypical’ cell projecting to the medial shell of the accumbens in Fig 8A3 and 8A4 and for an example ‘conventional’ cell projecting to the lateral shell in Fig 8B3 and 8B4. The newly recorded cells showed similar spike width and magnitude, rise and fall rates and similar responses to hyperpolarizing pulses as in [10] and illustrated in Fig 6. However, we noted slower spontaneous pacemaker rates in atypical DA neurons compared to our previous study, which might be due small differences in mouse strains, postsurgical delay after bead injections and calcium buffering in pipette solutions (for additional recordings with increased calcium buffering and in response to small (5–25 pA) depolarizations, see S2 Fig. Depolarization block was induced in both types of cells using a square current pulse in steps of 25 pA (Fig 8A5 and 8B5). As predicted by our model, an additional current pulse (75 pA for 200 ms) was not as effective in evoking an additional spike from depolarization block in the medial projecting atypical population compared to the lateral projecting conventional population. As with the model, the capacitive current was estimated by plotting dV/dt (Fig 8A6 and 8B6).

**Fig 9:**
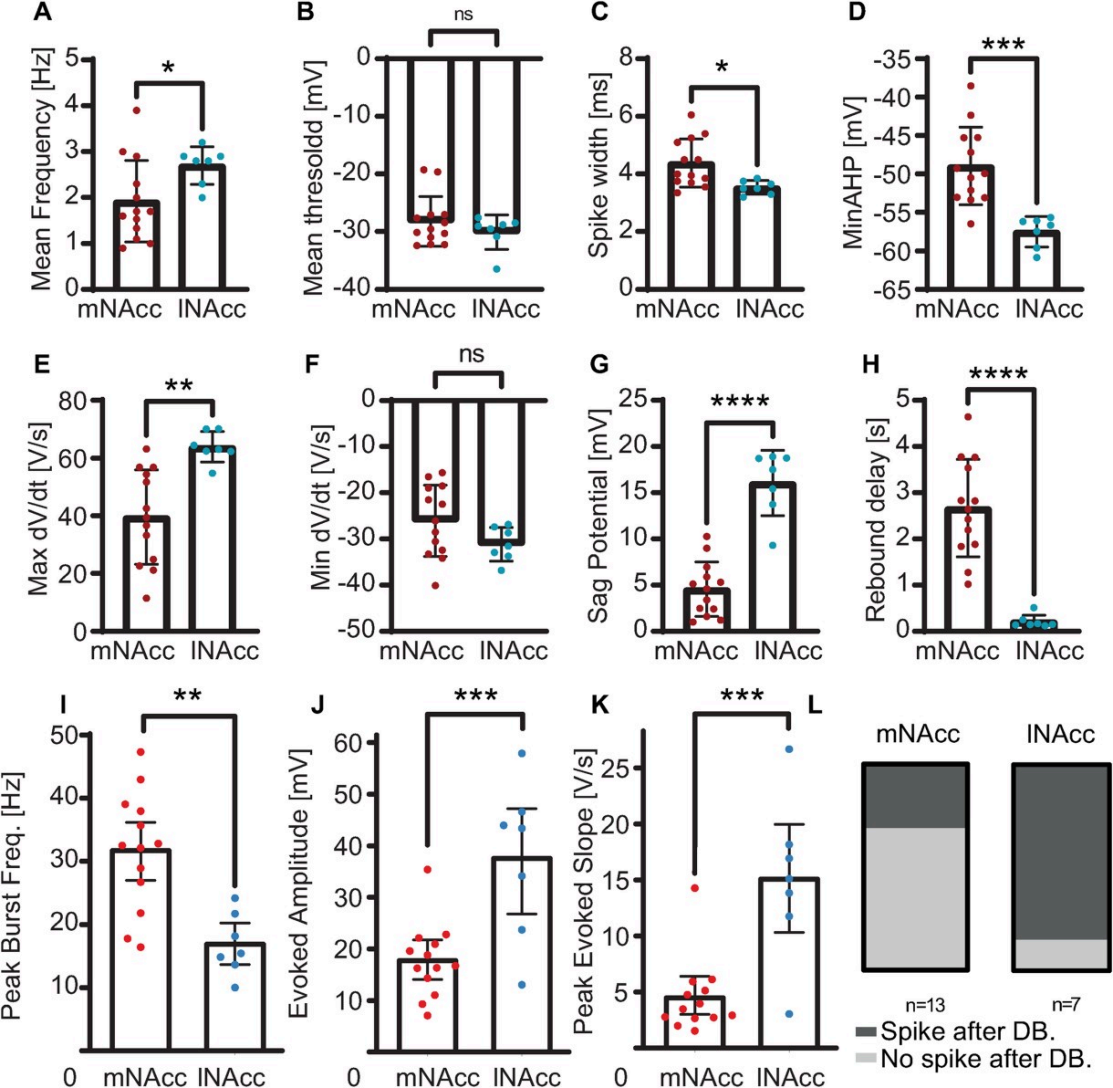
Statistical Summary of Experimental Results. **A.** Pacemaker frequency. **B.** Spike threshold as defined by 5 mV/ms rise rate during pacing. **C.** Spike width at threshold from B. **D.** Peak rate of depolarization during pacing (V/s) **E.** Peak rate of repolarization during pacing. **F.** Minimum AHP (mV) during pacing. **G.** Sag potential following hyperpolarization to -80 mV. **H.** Rebound delay (s) following hyperpolarization from G. **I.** Peak frequency (Hz) at first observed entry into depolarization block (25 pA intervals). **J.** Amplitude of evoked response to 75 pA, 200 ms pulse at 1.5 seconds into depolarizing pulse from I. **K.** Peak dV/dt for evoked response to 200 ms pulse from J. **L.** All lateral projecting, but only some medial projecting cells clear 5 mV/ms spike threshold. N = 13 for medial shell projecting (red), N = 7 for lateral shell projecting (blue).

Fig 8 contains experimental examples for comparison with the model. Statistics for each these properties over all recorded cells are shown in Table 2 and Fig 9. First, we compared our electrophysiological results to the previous study that first identified the atypical population as distinct from the conventional one [10] based on projection target. While the model cells in Fig 7 fall within the range observed for recorded cells, the average firing rates for medial projecting cells was found to be lower than lateral projecting cells (Fig 9A), in contrast to [10] (see Table 2 and Discussion). Overall, our current data set did reproduce most of the previously identified electrophysiological differences between atypical and conventional DA neurons with one exception. The finding of slower average baseline firing rates for atypical compared to convention DA neurons, might be attributable to the different pipette solution. However, the highly significant differences in the respective biophysical fingerprints between atypical and conventional DA neurons were robust across the two studies (see Table 2). This included significant differences in action potential width (Fig 9C), AHP minimum (Fig 9D), maximum dV/dt (Fig 9E), sag amplitude (Fig 9G) and rebound delays (Fig 9H),

**Table 2:** Comparison with previous study.

	Lammel et al 2008 [10]			This Study		
	Atyp.	Conv.	P-Value	Atyp.	Conv.	P-Value
Frequency (Hz)	5.6	2.3	P<0.01	1.9	2.7	P = 0.04
Sag Amp.* (mV)	4.1	14.4	P<0.01	4.5	16.1	P<0.0001
Rebound delay* (s)	2.5	0.9	P<0.01	2.7	0.21	P = 0.0001
AP Width (ms)	4.5	3.2	P<0.01	4.4	3.5	P = 0.018
AHP Minimum (mV)	-41.7	-56.9	P<0.01	-49.0	-57.5	P = 0.005
Max dV/dt (V/s)	24.4	86.5	P<0.01	39.6	63.9	P = 0.0014

Fig 9 also presents summary statistics showing that the maximum transient frequency prior to entry into depolarization block was significantly higher in the atypical population (Fig 9I). A spike could be evoked in the medial projecting cells in about two thirds of the medial projecting cells compared to all of the lateral projecting cells (Fig 9L); the evoked spikes, if any, had a significantly smaller amplitude (Fig 9J) and a slower rate of rise (Fig 9K) than the lateral projecting cells, consistent with a lower availability of sodium channels in the atypical, medial shell projecting cells under these conditions.

Since the atypical model was calibrated using a faster baseline spontaneous firing rate than the conventional cells, whereas the current study did not find that to be the case, we ran additional simulations to show that the basal frequency had no effect on predicted responses to depolarization block. For the atypical model, an increase in g_SK_ that deepens the AHP from -51 mV to -54mV and a slight decrease in the sodium leak conductance (from 3 to 2.75 μS/cm^2^, equivalent to 12.5 pA at -50 mV and consistent with the experimental manipulations in S2 Fig) lowers the basal frequency of the atypical model to 2.2 Hz enhances changing the ability fire at faster frequencies prior to depolarization block, but did not change the gradual entry into depolarization block, nor the failure of an additional depolarization to evoke a spike (S3 Fig). These modelling results are similar to our new experimental data where injection of small-depolarizing currents in the range of 10–20 pA enhanced the pacemaker rate of atypical DA neurons into the previously reported range (S2 Fig). Similarly, increasing the frequency of the conventional model to 2.7 Hz by increasing g_LNa_ also does not change the depolarization block features of an abrupt failure at slower frequencies that preserves the ability of a subsequent depolarization to evoke a spike (S3 Fig). Thus, we do not have a single canonical model for each population, but a range of models with a range of baseline frequencies. However, the essential and robust differences between the two populations lie in the long-term inactivation characteristics of the sodium channel and the strength of the spiking conductances. Also, confidence in the ability of the model to characterize the effect of the SK is increased by the ability of the model (S4 Fig) to capture the experimentally observed response to SK channel block as in Fig 4A of [37], including the after-depolarizing potential ADP that is revealed.

## Discussion

### Model predictions

We have demonstrated that the differences in observed maximal firing rates and entry into depolarization block are consistent with differences in the long-term inactivation of sodium channels between conventional and atypical DA neurons. The model was simplified as much as possible while retaining the ability to account for the data. We started with the model from [27] and removed one state and a few transitions. The model is phenomenological, and the key aspect of the model is that the Markov states can be separated into two pools based on time scale, one in which the channels can be quickly made available for the next spike (all states except I2) and one in which they cannot (I2). This separation allowed us to capture the basic features of the two populations, including the faster maximum frequency of the atypical population and the abrupt failure in the conventional population. Importantly, this modeling study also made novel predictions that were confirmed by the experimental portion of the study. We proposed that in accordance with our model 1) an additional depolarizing stimulus applied during depolarization block would more readily evoke an additional action potential in conventional compared to atypical DA neurons (see Figs 7–9). This proposal was confirmed by new recordings from identified atypical and conventional DA neurons in brains slices from adult mice. 2) The gradual failure at higher frequencies than the conventional will persist for atypical cells forced into depolarization block for a square pulse of current (see Figs 7–9). A further prediction is that the atypical population will exhibit less long-term inactivation of the sodium channel. While this has not been experimentally addressed in this study, it could be tested using cell attached or outside out patches with potassium and H channels blocked to isolate the Na^+^ current or via difference currents with and without saturating TTX, as in experiments performed on the conventional population [14,15].

### Limitations of single compartment models

The values of 15,000 μS/cm^2^ and 30,000 μS/cm^2^ for the sodium channel density in the atypical and conventional single compartment models are somewhat higher than the average conductance density of 7000 μS/cm^2^ (range 2900–18,000 μS/cm^2^) reported for rat SN [39]. These measurements were based on the estimated area of an outside-out patch and ignored inactivation by using only peak current measurements. As stated in the Methods, our Markov model cannot evoke the full conductance of the channel at any given time, peaking at ~50% occupancy [29] in the open state in response to voltage steps from -120 to 0 mV as in Fig 1D. Conductances were tuned primarily to match known [10] dV/dt vs V phase plots during pacing for both atypical and conventional populations. Other studies in rat SN [14,15] somata found lower values (1433 μS/cm^2^ and 2450 μS/cm^2^, respectively). A study in mice [40] obtained 500 uS/cm2 and 680 μS/cm^2^ in SN and VTA somata respectively, with values 5 to 10 times respectively higher in the axonal blebs. However, the absence of a separate axon from our model means that the characteristics of the sodium current, including the conductance and voltage dependence of activation are not only represent the soma, but also any contributions from the axon (and dendrites). Since the model does not include an axon initial segment or axon-bearing dendritic compartments, the shape of the action potential does not include separate contributions from these compartments [41]. The single compartment model is not able to make predictions regarding non-uniform entry into depolarization block between somatic and dendritic compartments.

Our single compartment model focuses on the contribution of intrinsic dynamics to the firing pattern and allows for insight into the underlying intrinsic mechanisms but cannot capture many nuances of synaptically driven spike patterning. For example, a recent study [42] showed that pedunculopontine nucleus synaptic input to proximal dendrites essentially by-passes the somatic compartment and can pattern axonal spiking in relatively arbitrary ways. Morphologically detailed models are required to address the full dynamics of the firing pattern *in vivo*.

### Comparison with prior models of limitation of the firing rate

The question of what limits the maximal firing rate of midbrain dopamine neurons *in vitro* was previously the subject of theoretical predictions by others [43,44]. These models represented the dopaminergic neuron as a set of electrically coupled oscillators with different natural frequencies, in which each frequency was determined by the surface area to volume ratio of the compartment, and the proximal dendrites play a primary role in setting the frequency of oscillation. Moreover, they predicted that the maximum frequency limitation was due to the rate of calcium accumulation and depletion in the proximal dendrites that could not support frequencies greater than 10 Hz. They further argued that if the smaller, distal dendritic compartments received sufficient NMDA receptor stimulation such that the distal dendritic oscillation overpowered the proximal one, then the frequency limitation could be exceeded. However, applying a virtual square pulse of NMDA conductance at the soma was sufficient to overcome the frequency limitation [45], thus the calcium dynamics in the larger proximal compartments do not limit the maximum frequency.

Our study provides a new explanation for the limitation of the firing rate in the conventional population, and for why the frequency in the atypical population does not have the same limitation. The mechanism relies on a Markov model of the sodium channel, with a long-term state that explains the slow recovery from inactivation of these channels as observed in conventional dopamine neurons [14,15]. The occupancy in the long-term inactivated state controls the maximum firing rate. This is because the interval between spikes has to be sufficiently long to recover from the amount of long-term inactivation induced by the previous spike, or spiking stops. Of course, other currents can speed or slow the maximum rate, but long-term inactivation of the sodium current is primarily responsible for the depolarization block that ultimately limits the frequency. The evidence for this mechanism is strengthened by the confirmation of the prediction that sodium channel availability is lower in the atypical population upon entry in depolarization block; the generally more abrupt failure in the conventional population preserves the ability to generate an action potential in response to an additional stimulus.

### Relationship to previous studies comparing conventional and atypical dopamine neurons

The first study to identify the atypical DA population in the VTA [46] found that these calbindin-positive DA neurons located in the VTA had faster pacemaker rates compared to conventional DA neurons in the SN. Importantly, both in standard whole-cell recordings with high calcium buffering (10 mM EGTA) and in perforated-patch recordings that preserve cell-intrinsic calcium signaling, atypical DA neurons–also characterized by low HCN channel densities and long rebound delays–discharged at fast rates of about 5 Hz (Fig 3 in [46] for whole cell recordings & Fig 10 in [46] for perforated patch). Similar results were found, with the added feature of defined axonal projection defined, for adult atypical and conventional DA neurons by [10]: 5.6 versus 2.3 Hz on average. In the current experimental study, we used two pipette solutions, which are likely to capture also the physiological differences in calcium handling between conventional and atypical DA neurons (0.1 mM EGTA and 1 mM BAPTA). In contrast to previous studies, we observed slower spontaneous pacemaker rates in atypical DA neurons with both solutions compared to our previous studies, However, other biophysical properties, including those tested explicitly in context with model predictions, were very similar to previous studies and robustly observed in both internal solutions. Other small differences in mouse strains and bead-labelling protocols might be responsible for the differences in spontaneous firing of the atypical DA neurons which could be compensated—experimentally and in silico—by adapting the depolarizing background current.

In the experimental part of the current study, we focused on projection-defined subgroups of atypical and conventional DA neurons. DA VTA neurons projecting to the medial shell of nucleus accumbens displayed atypical features (small sag, long rebound delay) while DA VTA/SNc neurons projecting to the lateral shell of nucleus accumbens possessed conventional features (large sag, short delay), both in accordance with our previous data [10]. However, using more physiological low calcium buffering patch-pipette solutions (0.1 mM EGTA), which we recently also evaluated *in vivo* [47] for both atypical and atypical DA neurons, we realized that spontaneous pacemaker frequencies were lower in atypical DA neurons (about 2 Hz), while their other signature properties such as low sag amplitude, long rebound delay and–most importantly in the context of this study–dynamic range of firing upon current injection were preserved. In contrast, pacemaker frequencies of conventional DA neurons were in the same range compared to our previous studies.

In any case, the baseline firing rate is not fundamental to the key differences between the atypical and conventional populations. A lesser tendency for Nav channels in the atypical population to enter the long-term inactivated state allows them to have a faster maximum frequency and thereby a greater dynamic range. Somewhat counterintuitively, the atypical population with its lesser tendency to enter the long-term inactivated state nonetheless depletes the available pool of sodium channels more thoroughly with its gradually tapering spikes. Therefore, the conventional population is more likely to produce a spike when it receives an additional depolarization while it is in depolarization block.

### Additional subpopulations identified by molecular profiling

Although the distinction between conventional and atypical electrophysiology may at the top of the hierarchy of subpopulation diversity [48], there are certainly other dimensions that further subdivide midbrain dopaminergic neurons. Molecular profiling of the Grp gene that encodes the neuropeptide gastrin-releasing peptide and the Neurod6 transcription factor involved in the differentiation of nervous system has revealed a more granular subpopulation structure [49]. A subset of atypical dopamine neurons express Grp+/Neurod6+, are located in the ventromedial VTA, and send projections to the medial shell of the nucleus accumbens. Grp-/Neurod6+ dopamine neurons constitute a subset of the VTA atypical subpopulation. A subset of the conventional neurons are Grp+/Neurod6- dopamine neurons located in the VTA and the ventromedial portion of the SNc that project selectively to the dorsomedial striatum rather than the dorsolateral striatum. Molecular profiling of the Cck gene that encodes the neuropeptide cholecystokinin and the Crhr1 gene encoding a corticotrophin-releasing hormone receptor revealed that VTA DA neurons projecting to the nucleus accumbens core mediate Pavlovian reward learning and selectively express Crhr1 whereas those projecting to the shell selectively express Cck and do not participate in Pavlovian reward learning [50].

### Comparison with previous models of depolarization block

Previous Hodgkin-Huxley (HH) type [21,22] models with multiplicative, independent state variables for slow and fast recovery from inactivation failed to fully capture the sharp failure in spike amplitude observed by [10,12]. The sharp entry into depolarization block is enabled by the effect of long-term inactivation on the bifurcation structure as described in the text for Figs 4, 7, and S1. Consistent with a previous study [51], the mechanism for hysteresis in dopamine neurons during a ramp response is supported by bistability between depolarization block and spiking in conventional cell models, allowing for both steady down ramps and the partial rescue of spiking with current steps. However, as explained the in results text for Fig 4, the atypical cell models do not have enough occupancy in the long-term inactivated state at their unstable “resting potential” in the presence of applied depolarizing currents to prevent regenerative activation of inward currents. The lack of a stable resting potential near the firing threshold, even with saturated I2, corresponds exactly to a lack of the recessed subcritical Hopf that allows for the sharp entry into depolarization block. In these cells, continued depolarization block in the down ramp is caused by both an initial overshoot of long-term inactivation from the initial failure, and a sufficiently slow recovery from inactivation to remain below the available sodium pool to resume firing at the instantaneous driving current.

The previous paragraph refers to the bifurcation that terminates spiking; there is also a bifurcation by which spiking is initiated that controls whether neurons have a resonant frequency near threshold. Some neurons can fire arbitrarily slowly because the steady state current in the range of voltages traversed is always inward. However, this is not a sufficient condition. The AHP of the action potential must be sufficiently hyperpolarized to reach or overshoot the resting potential of the membrane at the exact value of applied current at which the resting potential becomes unstable and is replaced by pacemaking. If the AHP meets this condition, then pacemaking can be arbitrarily slow and the mathematical term for the bifurcation is a saddle node on an invariant limit cycle. If not, then the stable rest potential is bistable with pacemaking for some range of applied current, there is an abrupt onset of pacemaking at a minimum threshold frequency, and the mathematical name for the bifurcation is a saddle node not on an invariant limit cycle [52,53]. The shallower AHP of the atypical population makes them more likely to exhibit a saddle node not on an invariant limit cycle bifurcation (S1A1 Fig) at the onset of spiking compared to the conventional (S1B1 Fig) which are more likely to exhibit a saddle node bifurcation in an invariant limit cycle).

Here we studied the principles that limit the maximum rate and mode of entry into depolarization block in the context of midbrain dopamine neurons, but they are likely quite general. For example, CA1 pyramidal neurons cannot sustain firing above 30–50 Hz and enter depolarization block with a sharp failure of action potential amplitude [54]. The same study showed that a one-compartment HH model with only a single inactivation variable went into depolarization block very gradually; a sharp failure was achieved in a small parametric regime with a multi-compartmental model but no mention was made of the mode of failure.

### Implications for dopaminergic signaling and disease

*In vivo* pacemaking is preserved in most identified dopamine neurons under isoflurane anesthesia [47] as well as in awake behaving animals [55]. Dopamine neurons are not simply leaky integrators processing their synaptic inputs to determine threshold crossings; instead, the intrinsic properties of dopamine neurons critically shape their patterns of electrical activity *in vivo*. However, network interactions are also an important factor.

There is accumulating evidence that different subpopulations of midbrain dopamine neurons are differentially affected by pathological processes, and the subpopulation distinctions are likely more granular than simply atypical versus conventional. For example, hyperactivity in mesolimbic-projecting subpopulations has been selectively implicated depression-like behaviors [56–58]. Similarly, an associative, dorsomedial-striatal-projecting subpopulation is selectively dysregulated in schizophrenia [6,59]. Better understanding of the distinct intrinsic electrophysiological properties of identified subpopulations may be useful in designing therapies targeted to specific subpopulations of dopamine neurons.

## Methods

### Ethics statement

All experimental procedures were approved by the German Regional Council of Darmstadt (TVA 54-19c20/15-F40/30).

### Single compartment model

**Fig 10:**
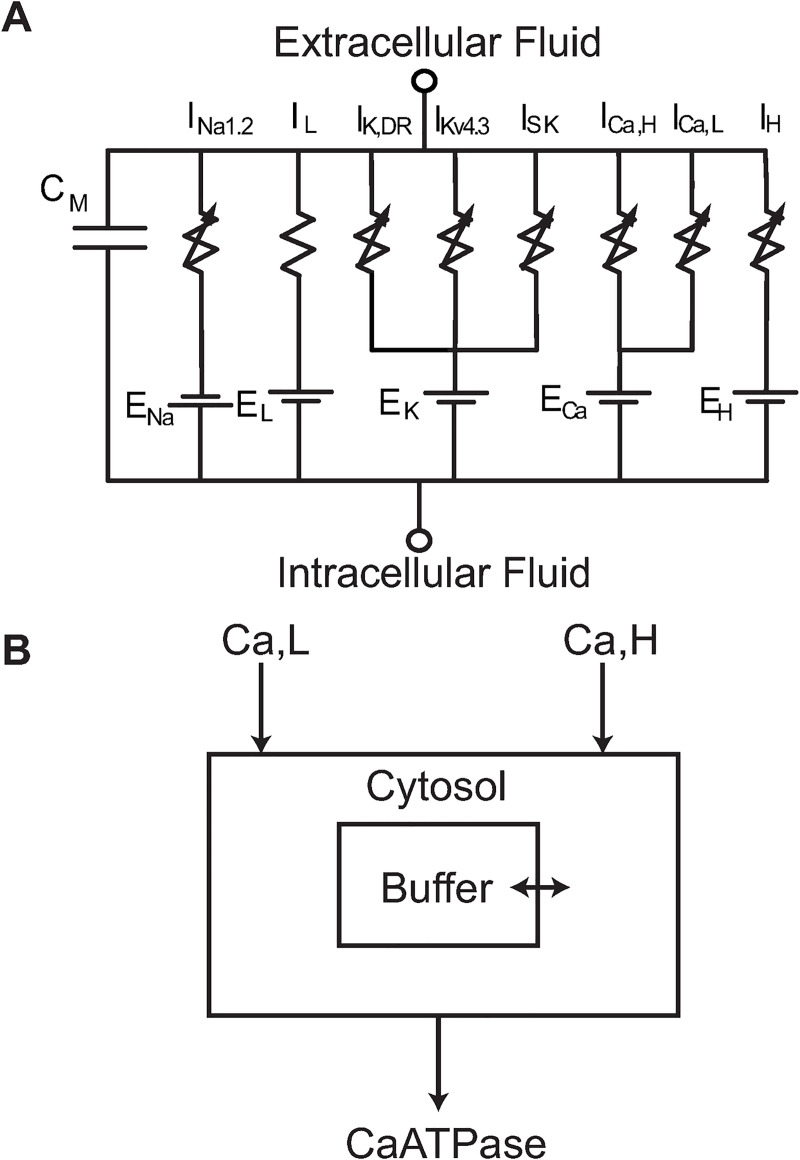
Single Compartment Dopamine Neuron Model. **A**. Circuit diagram of one compartment model with leak, sodium (Na_V_ 1.2), delayed rectifier potassium, A-type potassium (Kv4.3), Calcium activated potassium (SK), L and N type calcium channels, and an HCN channel. **B**. Calcium balance. Calcium is dynamically buffered using the mechanisms from [62]. The volume was restricted to that of a single submembrane shell (0.5 μm thick) to approximate a dendritic calcium response.

Here we focused on the key factors contributing to limiting the firing rate in midbrain dopamine neurons. For clarity and simplicity, we illustrated these mechanisms in a single compartment model. We included a leak current (I_L_), a voltage-gated sodium current (I_NaV1.2_), a delayed rectifier (I_K,DR_), the transient outward current (I_Kv4.3_), a calcium-activated SK channel (I_SK_), a fast inactivating, high threshold (I_Ca,H_) and a slowly inactivating, low threshold calcium current (I_Ca,L_), and the hyperpolarization-activated, nonspecific cation H current (I_H_) in parallel with a membrane capacitance (C_M_) (Fig 10A). The high and low threshold calcium channels group qualitatively similar channels (N/P/Q/R, and L/T) together and contribute to a single shared Ca^2+^ pool with dynamic buffering (Fig 10B). Certain parameters (highlighted in bold below) are known to be different between the two populations, as described in the Introduction, and others were inferred to be different from the fact that the AHP is shallower in the atypical versus conventional population. These putative differences are given in Table 1, for a first attempt at modeling the differences between the two populations. The model was implemented in NEURON [60] and is freely available at http://modeldb.yale.edu/266814.


$$C\frac{dV}{dt}={-I}_{Na1.2}-I_{Kdr}-I_{Kv4.4}-I_{SK}-I_{CaL}-I_{CaH}-I_{L}-I_{H}+\frac{I_{stim}}{2\pi \;d\;L}$$ (1)

The voltage gated sodium channel (NaV1.2) is modelled with a Markov scheme adapted from [27] consisting of 5 states (1 open, 2 closed, 2 inactivated) arranged as shown in Fig 10A. The current is linear with respect to the occupancy of the open state:

$$I_{NaV}=g_{Na1.2}\;O_{1}(V-50)$$ (2)

Where the maximal conductance, g_Na1.2_, varies between atypical and conventional models (Table 1). State dynamics are governed by linear transitions between states with voltage dependent rate functions. These rate equations, except for R_O1-I1_, are of the form:

$$R_{a‐b}=R_{max}Boltz(V,V_{H},V_{S})$$ (3)

where ‘Boltz’ is a Boltzmann sigmoid function:

$$Boltz(a,b,c)=\frac{1}{1+exp(-\left(a-b\right)/c)}$$ (4)

R_O1-I1_ is the sum of two Boltzmann functions. The values for R_max_, V_H_, V_S_ for each transition are given in Table 3. Kinetics were altered from [27] to increase the occupancy of the open state during spiking and depolarizing pulses to fractions more consistent with the literature.

**Table 3:** Parameters for Markov sodium channel transition rates. R_O1-I1_ is the sum of the two Boltzmann functions respectively parameterized by the first and 2^nd^ values. The maximal rate for the I1-I2 transition varies between populations (see Table 1).

	C1-C2	C2-C1	C2-O1	O1-C2	O1-I1	C2-I1	I1-C1	C1-I1	I1-I2	I2-I1
R_max_	12	0.5	14	4	0.5, 2.5	0.06	0.2	0.2	**C**_**I1I2**_	3.6e-3
V_H_	-8	-50	0	-48	-42, 10	-65	-65	-65	-25	-50
V_S_	10	-9	6	-9	-12, 12	11	-10	11	5	-10

All other channels follow a Hodgkin Huxley [26] form with state kinetics given by:

$$\frac{dx}{dt}=\frac{x_{\infty }(V)-x}{\tau_{x}(V)}$$ (5)

The delayed rectifier potassium channel is adapted from [61]. The original sigmoid was modified to resemble a skewed Gaussian such that the channel rapidly deactivates at hyperpolarized voltages. This aspect of K_dr_ is required to capture the sensitivity of AHP depth to simulated SK channel blockers (S4 Fig). The delayed rectifier current and kinetics are given by:

$$I_{Kdr}=g_{Kdr}\;n^{3}\;(V+90)$$ (6)

$$n_{\infty }(V)=Boltz(V,-20,12)$$ (7)

$$\tau_{n}(V)=1+5\;\exp(‐{\left[\log(1+0.05\;(V+40)/0.05\right]}^{2}/300)\;\mathrm{for}\;V>‐40$$

$$\tau_{n}(V)=2+4\;\exp\;\left(-\frac{{\left(V+40\right)}^{2}}{8}\right)\;\mathrm{for}\;V\leq ‐40$$ (8)

Where g_Kdr_ varies between conventional and atypical models (see Table 1).

The Kv4.3 (A-type) potassium channel is adapted from [35] and is required to produce the pronounced linear rebound delays that are characteristic of atypical dopamine neurons. Its current and kinetics are given by:

$$I_{Kv4.3}=g_{Kv4}p^{3}q(V+90)$$ (9)

$$p_{\infty }(V)=Boltz(V,-35,7)$$ (10)

$$q_{\infty }(V)=Boltz(V,-61,-4.5)$$ (11)

$$\tau_{p}(V)=0.1029+0.483\;Boltz(V,-56.7,-6.22)$$ (12)

$$\tau_{q}(V)=\tau_{Kv4}$$ (13)

In both models, gKv4 = 450 μS/cm^2^, τ_Kv4_ is constant with respect to voltage; but varies between populations (see Table 1). The small conductance calcium activated potassium channel (SK) is adapted from [61] with current and kinetics given by:

$$I_{SK}=g_{SK}\;s\;(V+90)$$ (14)

$$s_{\infty }(\left[{Ca}^{2+}\right])=\frac{1}{\left(1+{\left(\frac{\left[{Ca}^{2+}\right]}{190\;nm}\right)}^{-4}\right)}$$ (15)

$$\frac{ds}{dt}=\frac{s_{\infty }(\left[{Ca}^{2+}\right])-s}{5.0\;ms}$$ (16)

In real dopamine neurons, the dynamics of the SK current are largely driven by dendritic calcium dynamics [43,61]. In order to approximate these dynamics in a one compartment model that simulates somatic recordings, the calcium balance (Fig 10B) on the free calcium concentration that activates I_SK_ was performed on the outer 0.5 μm shell of a uniform 5 μm diameter compartment with no radial diffusion. Calcium is dynamically buffered using a mechanism adapted from [62]. Calcium is removed via a non-electrogenic calcium pump (Ca-ATPase). The calcium dynamics were calibrated to evoke an AHP depth of 15 mV with a 100 ms duration, consistent with the effect of SK-blockers on conventional cells [38]. The calcium dynamics are given by:

$$\frac{d[{Ca}^{2+}]}{dt}=\frac{d}{2\;F}\frac{\left(I_{Ca}+I_{CaPump}\right)}{d-0.5}$$ (17)

$$I_{CaPump}=\frac{1.91}{1+\frac{500\;(nM)}{\left[{Ca}^{2+}\right]}}$$ (18)

$$\left[{Ca}^{2+}]+[Bu]\underset{k1,k2}{\Leftrightarrow }[CaBu\right]$$ (19)

Where k1 = 100 mM/ms, k2 = 0.1/ms, d = 5 microns, and F is Faraday’s constant. A single low threshold calcium channel model was included to capture the effects of the L and T type calcium channels. The L-type calcium model is adapted from [61] with the addition of partial inactivation fit to data from whole cell recordings of SNc DA neurons [63].


$$I_{CaL}=g_{CaL}\;m_{L}(0.4+0.6h_{L})(V-60)$$ (20)

$$m_{L\infty }(V)=Boltz(V,-30,5)$$ (21)

$$h_{L\infty }(V)=Boltz(V,-50,-2)$$ (22)

$$\tau_{mL}(V)=0.3+9\;\exp(-{\left[V-70\right]}^{2}/25)$$ (23)

$$\tau_{hL}(V)=100+100\;Boltz(V,-30,5)$$ (24)

With g_CaL_ = 5 μS/cm^2^. While the T-type calcium channel has significant effect in response to rapid recovery from hyper polarizations in conventional VTA/SNc DA cells, [64,65] the channel is largely inactive during both prolonged depolarizations. As its current profile during pacing is qualitatively like that of an inactivating L-type current, the non-rebound effects of this channel were subsumed into an increased inactivating component of the L-type channel. A single calcium channel model for rapid, high threshold calcium entry was used to describe the N, P/Q, and R calcium channels. The current and kinetics of this channel are adapted from a model of an N-type calcium channel in retinal ganglion cells given by:

$${{I_{CaH}=g}_{CaH}m_{H}^{2}h}_{H}(V-60)$$ (25)

where g_CaH_ = 50 μS/cm^2^ and:

$$m_{H\infty }(V)=a/(a+b)$$ (26)

$$\tau_{mH}(V)=1/(a+b)$$ (27)

$$h_{H\infty }(V)=c/(c+d)$$ (28)

$$\tau_{hH}(V)=1/(c+d)$$ (29)

where:

$$a=0.1\frac{V-20}{\left[1-\exp\left(\frac{V-20}{10}\right)\right]}$$ (30)

$$b=0.4\;\exp\;\left(‐\frac{V+25}{18}\right)$$ (31)

$$c=0.01\;\exp\;\left(-\frac{V+50}{10}\right)$$ (32)

$$d=0.1\;\exp\;\left(-\frac{V+17}{17}\right)$$ (33)

The h-current is adapted from (35) with current and kinetics given by:

$$I_{H}=gH\;m_{H}(V-40)$$ (34)

$$m_{H}(V)=Boltz(V,-75,-5)$$ (35)

$$\tau_{H}(V)=3/\left(2e‐8\;\exp\left(‐V/10.2\right)+7.6e‐3\;Boltz(V,-10,100)\right)$$ (36)

The h-current is present only in conventional models (see Table 1). Lastly, the model cell contains passive potassium and sodium leak currents with conductances of 4 and 3 μS/cm^2^, respectively.

In all manipulations in Figs 3 and 4, the minimum peak ramp amplitude (in 5 pA steps) that resulted in either a termination of oscillations prior to the current peak or a terminal oscillation less than 20% that of the initial amplitude was deemed to be the threshold for entry into depolarization block.

In the bifurcation analysis of the models, fixed points were determined via a quasi-Newton search and the local Jacobian was determined numerically. Unstable limit cycles and bifurcation types are approximate due to high dimension (D = 19) of the system. Hopf bifurcations were identified by a change in sign of the largest Jacobian eigenvalue and the creation/destruction of at least one complex pair of eigenvalues.

### Experimental methods

We used retrograde labeling in combination with whole-cell in-vitro patch-clamp recordings from male 8–12 weeks old C57BL/6N mice (total of 6 mice, 3 for each projection site). Recorded cells were stained with 0.1% Neurobiotin. After post-hoc immunocytochemical (TH+ RB+ NB+) identification of recorded neurons as dopaminergic and verification of accumbens injection sites, we included n = 20 validated cells for further analysis: DA NAcc lateral shell projecting (conventional): n = 7, DA NAcc medial shell projecting (atypical): n = 13.

We compared our current experimental data on the electrophysiological properties of the two different subpopulations to a previously published data set [10]. The experimental conditions between these studies were similar but not identical. Compared to [10], we now use an improved pipette solution with 135 mM K-gluconate, 5 mM KCl, 10 mM HEPES, 0.1 mM EGTA, 5 mM MgCl2, 75 μM CaCl_2_, 5 mM ATP, 1 mM GTP, 0.1% Neurobiotin, pH 7.35 (290–300 mOsm). The improved pipette solution resulted in physiological levels of free calcium concentration (~ 80 nM, see [8]). For synaptic blockers, Lammel at al. 2008 blocked AMPA/NMDA and GABA-A synaptic receptors with 20 μM CNQX and 10 μM gabazine respectively, whereas in the present study synaptic channels were blocked with 12.5 μM CNQX, 4 μM gabazine, and 10 μM of the NMDA specific blocker DL-AP5.

## Supporting information

S1 Fig Bifurcation Structure of Atypical and Conventional Models. **A1.** Bifurcation diagram for atypical model repeated from Fig 4D. Type 2 firing in the atypical model can potentially result from a saddle node (SN) bifurcation not on an invariant circle [52,53], if the AHP is too shallow for the action potential to reach or overshoot the saddle node. Consistent with failure of spiking at small amplitude at large DC, ramp currents, oscillations terminate via a super critical Hopf (SupH). **A2**. Demonstration of bistability of pacing and quiescence near the SN bifurcation. **A3.** Demonstration of irregular firing in an atypical model within region (25 pA) between period doubling/period halving bifurcations (PD/PH in A1). It is unclear if the dynamics are chaotic, this is a low dimensional approximate representation of a high dimensional system. **B1.** Bifurcation diagram for conventional model. Dynamics progress from a stable fixed point to a saddle node on an invariant limit cycle (SNIC) at -8 pA producing type 1 excitability. Bistability exists between pacing and depolarization block in the current range between the subcritical Hopf at 20 pA and the saddle node of periodics (SNP) at 45 pA. **B2.** Demonstration of bistability between pacing and depolarization block. As the basin of attraction of this state is extremely narrow, this region can generally only be reached by slowly decreasing the bias current from a state of monostable depolarization. A transient perturbation (current trace at bottom) causes a transition from depolarization block to pacing. **C.** Steady state occupancy in I2 state for atypical (black) and conventional (red) models.
(EPS)

S2 Fig New Experimental Data Confirming the Baseline Frequency does not affect other attributes of the atypical population. Small amounts of added current were used to gauge whether baseline frequency affected the other atypical attributes. **A1**. Representative example of spontaneous firing. **A2**. Single action potential waveform. **A3**. Phase plane representation. **B**. Representative example of response to hyperpolarization. **C1**. Representative entry into depolarization block and failure of second pulse to evoke a large amplitude spike. **C2**. dV/dt to approximate somatic charging currents. **D**. Dependence of frequency on injected current. This figure utilized 1 mM BAPTA in the pipette compared to 0.1 mM EGTA in Fig 8 in order to reduce the AHP to make the waveforms more comparable to the previous study [10].
(EPS)

S3 Fig Spontaneous Frequency Does Not Control Model Responses to Depolarization Block. **A**. Increasing g_SK_ from 20 to 50 μS/cm^2^ and decreasing g_LNa_ from 3 μS/cm^2^ to 2.9 μS/cm^2^ in the atypical model decreases basal firing rate to 2.2 Hz. AHP depth is increased by 3 mV, consistent with deeper AHP depths in recorded atypical cells in this study relative to those recorded previously in [10]. Consistent with the lack of effect of elevated SK channels on the response to ramp currents (Fig 3C) these changes had no significant effect on entry into depolarization block. **B.** Increasing g_LNa_ from 3 to 3.75 μS/cm^2^ increases the firing rate of the conventional model from 1.2 to 2.7 Hz, consistent with recordings from lateral shell projecting recordings in the present study. This change also has no observed effect on entry into depolarization block.
(EPS)

S4 Fig Predicted Effects of Apamin on Conventional Model. **A.** Blockade of SK channel produces an after depolarization (ADP) consistent with the literature [66]. **B1**. Subthreshold voltage over 100 ms post spike initiation. **B2**. After depolarization terminates with saturation of Kv4 (A type conductance) **B3**. A-Type conductance begins in depleted state (instant vs steady) due to accumulated inactivation during prior depolarization. **C1**. Subthreshold voltage over 500 ms following action potential. **C2**. Sodium inactivation is initially high following action potential, but slowly depletes. **C3**. Sodium current initially increases but is unable to activate regeneratively due to the depleted pool. Sodium current then declines in magnitude due to repolarization driven by A-type current until recovery from inactivation produces sufficient sodium window current to overwhelm the saturated potassium currents. Three times scales are required for the ADP. The first minimum, which is the trough of the AHP, occurs due to rapid de-activation of the delayed rectifier.The depolarization that follows results from the currents that drive pacemaking. The maximum occurs due to the second time scale, the recovery from inactivation of Kv4. The next minimum results from the slowest process, recovery from long-term inactivation of the sodium channel, which provides sufficient window current to overcome the now saturated Kv4 channel. A small hyperpolarizing stimulus current (5 pA) was required to unmask the ADP in this instance.
(EPS)
